# Evolution of *Salmonella Typhi* outer membrane protein-specific T and B cell responses in humans following oral Ty21a vaccination: A randomized clinical trial

**DOI:** 10.1371/journal.pone.0178669

**Published:** 2017-06-01

**Authors:** Juan Manuel Carreño, Christian Perez-Shibayama, Cristina Gil-Cruz, Constantino Lopez-Macias, Pietro Vernazza, Burkhard Ludewig, Werner C. Albrich

**Affiliations:** 1 Institute of Immunobiology, Kantonsspital St. Gallen, St. Gallen, Switzerland; 2 Medical Research Unit on Immunochemistry (UIMIQ), Specialties Hospital, National Medical Centre “Siglo XXI”, Mexican Social Security Institute (IMSS), Mexico City, Mexico; 3 Division of Infectious Diseases and Hospital Epidemiology, Kantonsspital St. Gallen, St. Gallen, Switzerland; Public Health England, UNITED KINGDOM

## Abstract

Vaccination against complex pathogens such as typhoidal and non-typhoidal *Salmonella* requires the concerted action of different immune effector mechanisms. Outer membrane proteins (Omps) of *Salmonella* Typhi are potent immunogens, which elicit long-lasting and protective immunity. Here, we followed the evolution of *S*. Typhi OmpC and F-specific T and B cell responses in healthy volunteers after vaccination with the vaccine strain Ty21a. To follow humoral and cellular immune responses, pre- and post-vaccination samples (PBMC, serum and stool) collected from 15 vaccinated and 5 non-vaccinated individuals. Immunoglobulin levels were assessed in peripheral blood by enzyme-linked immunosorbent assay. B cell and T cell activation were analyzed by flow cytometry. We observed a significant increase of circulating antibody-secreting cells and maximal Omp-specific serum IgG titers at day 25 post vaccination, while IgA titers in stool peaked at day 60. Likewise, Omp-specific CD4^+^ T cells in peripheral blood showed the highest expansion at day 60 post vaccination, concomitant with a significant increase in IFN-γ and TNFα production. These results indicate that *S*. Typhi Omp-specific B cell responses and polyfunctional CD4^+^ T cell responses evolve over a period of at least two months after application of the live attenuated vaccine. Moreover, these findings underscore the potential of *S*. Typhi Omps as subunit vaccine components.

**Trial registration:** ISRCTN18360696

## Introduction

*Salmonella* enterica serovar Typhi (*S*. Typhi) is an orally transmitted bacterial pathogen that infects only humans. Protective immune responses against this pathogen include the interaction of innate and adaptive immune mechanisms [1, 2]. Due to the host restriction, studies on disease pathogenesis and immune protection in the natural host are limited [3]. The Ty21a live attenuated vaccine strain represents a valuable model for studying immune responses that develop during infection with typhoidal *Salmonella* [3, 4]. Indeed, several studies have described the induction of humoral and cellular immune responses against different components of Ty21a after vaccination of healthy volunteers [5–11]. For example, IgM, IgG and IgA antibodies against membrane proteins can be detected in the plasma of healthy individuals even before the vaccination. However, ex vivo cultures of lymphocytes obtained early after Ty21a vaccination revealed higher levels of specific antibodies in supernatants compared to controls indicating that systemic B cell responses are swiftly activated after encounter of Salmonella antigens [6]. Antibodies against the O-9,12 antigen, plasmablasts specific for lipopolysaccharide (LPS), and flagellin [6, 7, 12] can be detected in circulation as early as day 7 after vaccination. Examination of immune cell properties revealed that *S*. Typhi-specific T cells bear homing receptors for intestinal tissues [8, 11], while the induction effector memory CD8^+^ T cells by Ty21a vaccination [13] suggests that cytotoxic T cell responses could at least in part contribute to the protection exerted by the vaccine. Furthermore, cross-reactive cellular immune responses include multifunctional CD4^+^ and CD8^+^ T cells [8, 10, 14] underscoring the importance of multiple layers of immune mechanisms involved in the protection against this bacterial infection [15]. However, a broad range of immune responses directed against different target structures is not always advantageous for the host. For example, dysregulated B cell responses against LPS favor replication of non-typhoidal *Salmonella* in human immunodeficiency virus (HIV)-infected individuals leading to reduced bactericidal activity of antibodies against otherwise protective bacterial antigens [16]. Hence, it is important to analyze the development of immune responses directed against protective antigens such as flagellin [17] or outer membrane proteins (Omps) [16] in the context of complex antigenic exposure such as vaccination with a live attenuated pathogen.

Pore-forming Omps (also known as porins) represent important antigenic targets for an efficient response against *Salmonella*. The immune response against these proteins is shaped initially through the direct TLR-dependent stimulation of antigen presenting cells [18]. Subsequently, porin-specific CD4^+^ T cells promote protective antibody responses via production of IFN- γ and other cytokines [19]. Individuals recovering from typhoid fever possess circulating IgG and IgM antibodies against porins [20] and purified *S*. Typhi OmpC and F, which form the major Omp fractions of *S*. Typhi, and induce IgM and IgG bactericidal antibodies in mice and humans [19, 21]. A vaccine candidate based on purified *S*. Typhi OmpC and F has been tested in a clinical trial revealing safety and immunogenicity following subcutaneous application [22]. Currently, novel formulations of *S*. Typhi porins for oral application are under investigation using encapsulation into biocompatible copolymers of lactic and glycolic acid (PLGA) microparticles rendering the proteins resistant to gastric acids [23].

To follow the evolution of *S*. Typhi OmpC and F-specific T and B cell responses in the natural host, we vaccinated healthy volunteers with the live attenuated vaccine strain Ty21a [24]. We found a significant increase in antibody-secreting cells present in peripheral blood and maximal serum IgG titers at day 25 post vaccination. Interestingly, both IgA titers in stool and Omp-specific polyfunctional CD4^+^ T cells in peripheral blood peaked at day 60 indicating that *S*. Typhi Omp-specific adaptive immune responses evolve over a period of at least two months after vaccination.

## Materials and methods

### Subjects, vaccination and sample collection

**Fig 1:**
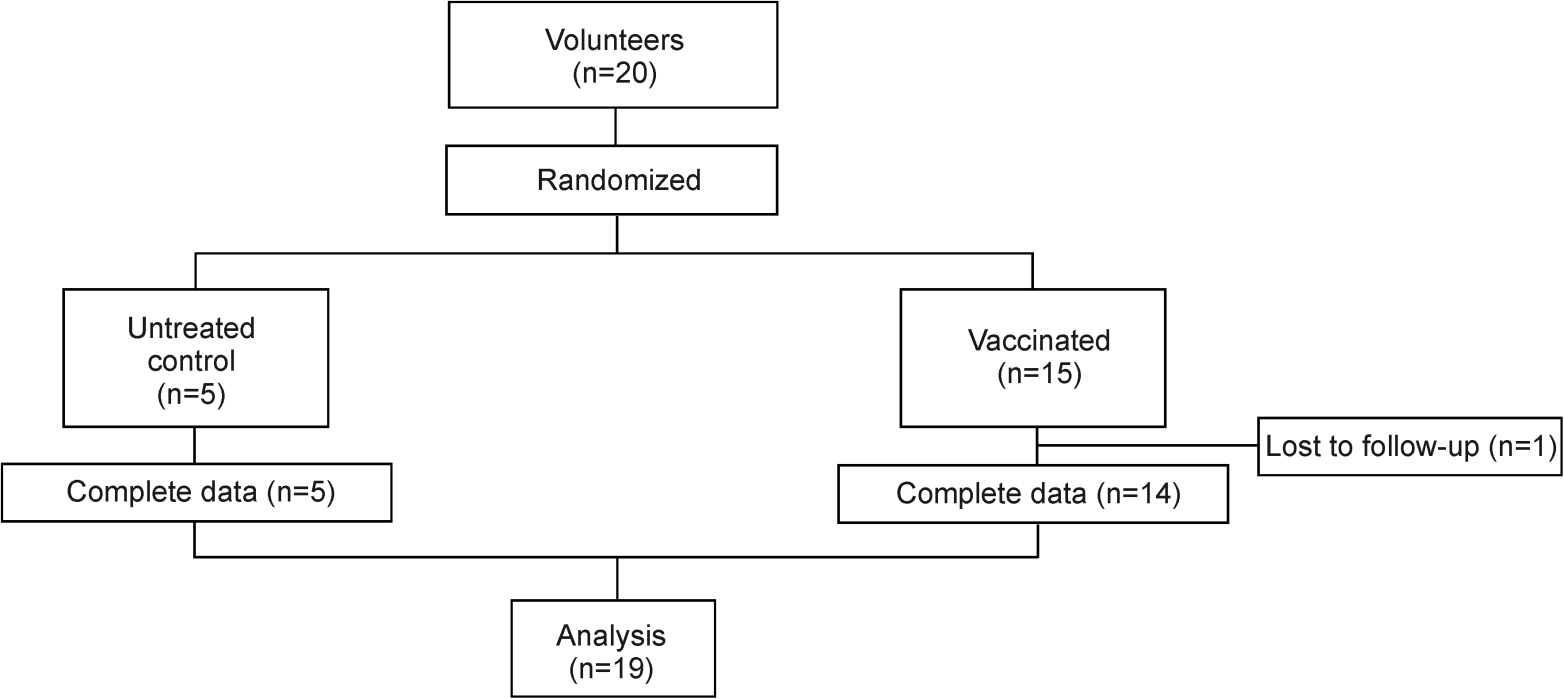
Study design. CONSORT flow diagram indicating the number of participants that have been included and have completed the study.

This open, interventional study was approved by the Ethics Commission of the Canton St. Gallen (EKSG 15/085) and has been published as ISRCTN18360696 (DOI 10.1186/ISRCTN18360696). Before enrolment, subjects were informed about the purpose of the study and written informed consent was obtained in compliance with local and global regulations (S1 File and S2 File). A physical examination and a medical questionnaire to ensure the health status of the participants were performed. Healthy volunteers enrolled in the study were 18–50 years old, without any previous vaccination against typhoid fever or infection with *S*. Typhi, with a negative test for HIV infection and a negative pregnancy test. In order to minimize bias block randomization was used. Randomization sequence was created with a 1:3 allocation using random block sizes of 4. Twenty volunteers were enrolled for the study from these, five volunteers were assigned as controls (all females, mean age 29.4 ± 6.4 years old), and 15 volunteers were assigned to the vaccinated group (10 females and 5 males, mean age 35.9 ± 8.4 years old) (Table 1). One volunteer of the vaccinated group abandoned the study before completion (Fig 1). Three enteric-coated capsules each containing 2–10×10^9^ live and 5–50×10^9^ dead lyophilized Ty21a bacteria (Crucell) [24] were administered orally every other day with lukewarm water; the subjects of the control group received no treatment. Blood and stool samples were collected before vaccination (day 0) and on days 11, 25 and 60 after application of the first vaccine dose (Table 1) and tested in a blinded fashion in regards to study group and tested in a blinded fashion in regards to study group assignment. PBMCs were isolated using Vacutainer CPT Ficoll tubes (Becton Dickinson) and frozen at -150°C in FCS with 10% DMSO (Sigma-Aldrich). Feces were mechanically disrupted, diluted (50% w/v) in a PBS solution containing 10% FCS and a protease inhibitor cocktail (1:500, Sigma-Aldrich) and centrifuged at 14,000 rpm at 4°C for 10 min. Clarified supernatants were collected and stored at -70°C until IgA measurement by ELISA. This study took place in the Kantonsspital St. Gallen, St. Gallen, Switzerland. The recruitment of the volunteers for the study started on June 15^th^, 2015, and ended on September 15^th^, 2015. The consort checklist of information to include when reporting a randomized trial is shown in S3 File.

**Table 1:** Study demographic details.

Group	Number	Gender (F/M)	Age (years)^#^	Vaccination
Vaccinated	15	(10/5)	35.9 ± 8.4	Vivotif
Control	5	5/0	29.4 ± 6.4	none

# Mean ± standard deviation.

### Production of S. Typhi Omps

OmpC and F proteins were purified from *S*. Typhi ATCC 9993 as previously described [21, 22]. LPS content was determined using the limulus amebocyte lysate (LAL) assay (Charles River Endosafe Laboratories), and all batches were found to be negative with a detection limit 0.2 ng LPS/mg protein. Western blot analysis using anti-LPS polyclonal sera confirmed that LPS was not detectable by these means.

### Detection of OmpC/F-specific antibodies

Antibody titers against S. Typhi OmpC/F in sera were determined as previously described [22]. Briefly, high-binding 96-well polystyrene plates (Corning) were coated with 10 μg/ml of the protein preparation in 0.1 M carbonate-bicarbonate buffer, pH 9.5. Plates were incubated for 1 h at 37°C and then overnight at 4°C. Before use, plates were washed three times with PBS containing 0.05% Tween-20 (PBS-T) (Sigma–Aldrich). Non-specific binding was blocked with 5% non-fat dry milk diluted in PBS (PBS-M) for 1 h at 37°C. After washing, sera were diluted 1:40 and stool extracts 1:2, both in PBS-M and twofold serial dilutions were added to the wells. Plates were incubated for 1 h at 37°C, followed by four washes with PBS-T. After 1 h of incubation at 37°C with peroxidase-conjugated rabbit anti-human IgG (1:10,000) or IgM (1:5000) antibody (in PBS-M, Jackson Immuno Research), four washes with PBS-T, ortho-phenylenediamine (0.5 mg/ml; Sigma) in 0.1 M citrate buffer, pH 5.6, containing 0.08% H_2_O_2_ was used to develop the reaction. Optical density was read at 492 nm using an automated ELISA plate reader (Tecan). Antibody titers are given as -log2 dilution × 40. Antibody titers were defined as the highest dilution of the sample at which the OD was higher than the mean ± 3 SD of the negative sample values.

### Identification of antibody secreting cells and activated B cells by flow cytometry

Activated B cells (ABC) and antibody secreting cells (ASC) were measured in blood as previously described [25]. Briefly, 10^6^ PBMCs were stained using the following: PerCP anti-human CD14, PerCP anti-human CD16 and APC-Cy7 anti-human CD20 from Biolegend; PerCP anti-human CD3, PE anti-human IgD, APC anti-human CD38, FITC anti-human CD19, and PECy7 anti-human CD71 from eBiosciences; the fixable viability stain 510 (e-Biosciences) was used to discriminate dead cells. Samples were stained for 30 min on ice with the viability dye, washed and stain for 20 min at 4°C with the required antibodies. Flow cytometric analysis was performed using a LSR-FORTESSA (Becton Dickinson). Data were analyzed using FlowJo software 10 (Tree Star, USA).

### In vitro stimulation and assessment CD4^+^ T cell responses

CD4^+^ T cell activation profile was assessed as previously described [26]. Briefly, 10^6^ PBMCs in RPMI 1640 medium containing 5% FCS, 1% penicillin-streptomycin were stimulated with 10 μg/ml of *S*. Typhi OmpC/F or 50 μg/ml of tetanus toxoid for 24h at 37°C. After *in vitro* stimulation, surface staining was performed and the frequency of CD4^+^ T cells and intracellular expression of CD40L, IFN- γ and TNF was assessed by flow cytometry using the following antibodies: PerCP/Cy5.5 anti-human CD3, PE/Cy7 anti-human CD4 and FITC anti-human CD154, PE anti-human IFN- γ and APC anti-human TNF (all from Biolegend); the fixable viability stain 780 (e-Biosciences) was used to discriminate dead cells. Samples were analyzed using a FACS Canto flow cytometer (Becton Dickinson), and data were analyzed using FlowJo software version 10 (Tree Star, USA).

### Statistical analysis

Statistical analyses were performed with Graphpad Prism 5.0 (GraphPad Software Inc. USA) using two tailed Student’s *t* test with Welch’s correction. Statistical analysis was performed using one way ANOVA with Dunnett’s multiple comparison test for comparisons between individuals of the same group at different time points (pre- versus post-vaccination) Statistical significance was defined as p < 0.05. Raw data is available as S4 File.

## Results

### Vaccine-induced B cell activation pattern in peripheral blood

**Fig 2:**
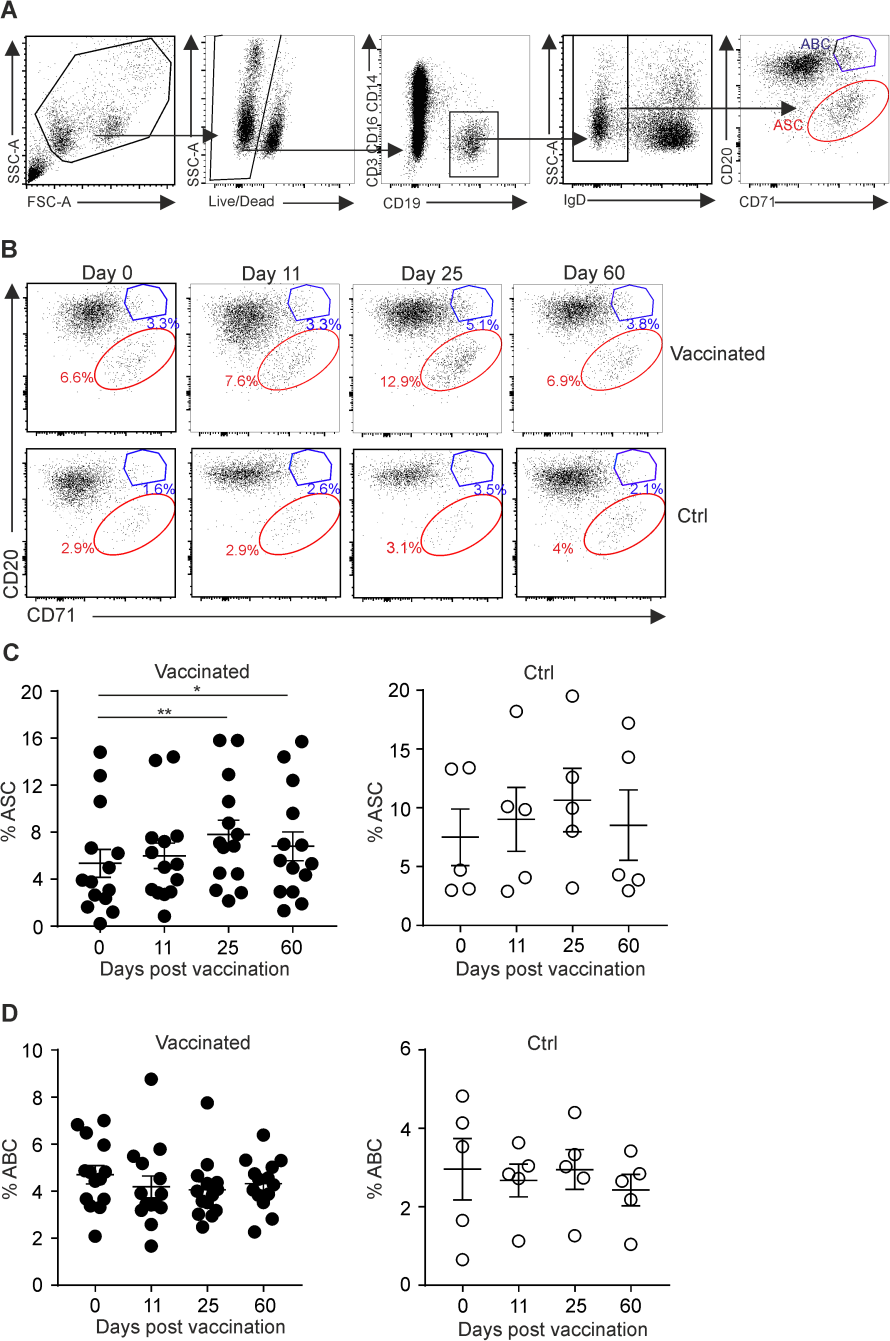
Identification of activated B cells and antibody secreting cells in peripheral blood following Ty21a vaccination. PBMCs were collected from vaccinated or control (Ctrl) subjects at the indicated time points and analysis of different B cell populations was performed by flow cytometry. (A) Gating strategy for FACS analysis of activated B cells (ABC, blue) or antibody secreting cells (ASC, red). (B) Representative dot plots showing the proportions of ABC (CD20^hi^ CD71^+^) and ASC (CD20^-^ CD71^+^) in vaccinated and Ctrl individuals at the indicated time points. (C and D). Proportions of ABC and ASC in vaccinated and Ctrl individuals at the indicated time points. Dots represent individual values; bars indicate mean ± SEM. Statistical analysis was performed using one way ANOVA with Dunnett’s multiple comparison test for comparisons between individuals of the same group at different time points (pre- versus post-vaccination) (*, P< 0.05; **, P< 0.01).

After infection or vaccination, pathogen-specific B cells proliferate and differentiate into antibody-secreting cells (ASCs) or memory B cells [27]. Following re-encounter with the pathogen, swift production of protective antibodies is secured by long-lived ASCs and rapid differentiation of memory B cells into ASCs [28, 29]. In order to assess how oral exposure to attenuated *S*. Typhi Ty21a affects B cell activation, we performed a flow cytometric analysis of PBMCs. Surface markers CD20 and CD71 on switched IgD^−^B cells were used to define either ASCs (CD20^–^ CD71^+^) or activated B cells (ABC, CD20^hi^ CD71^+^) (Fig 2A). This analysis revealed that the volunteers responded to the vaccination with a significant increase in circulating ASCs at days 25 and 60 after oral administration of Ty21a, a finding that was corroborated using staining for the ASC marker CD38 (S1A and S1B Fig). Notably, peripheral ASCs in non-vaccinated individuals were not significantly changed (Fig 2B and 2C and S1C Fig), while both groups of volunteers did not show substantial differences in ABC numbers throughout the course of the observation period (Fig 2D). These results indicate that oral exposure to *S*. Typhi Ty21a induces B cell activation and differentiation towards ASCs, which can be traced in peripheral blood of vaccinated individuals.

### Induction of OmpC/F-specific antibodies

**Fig 3:**
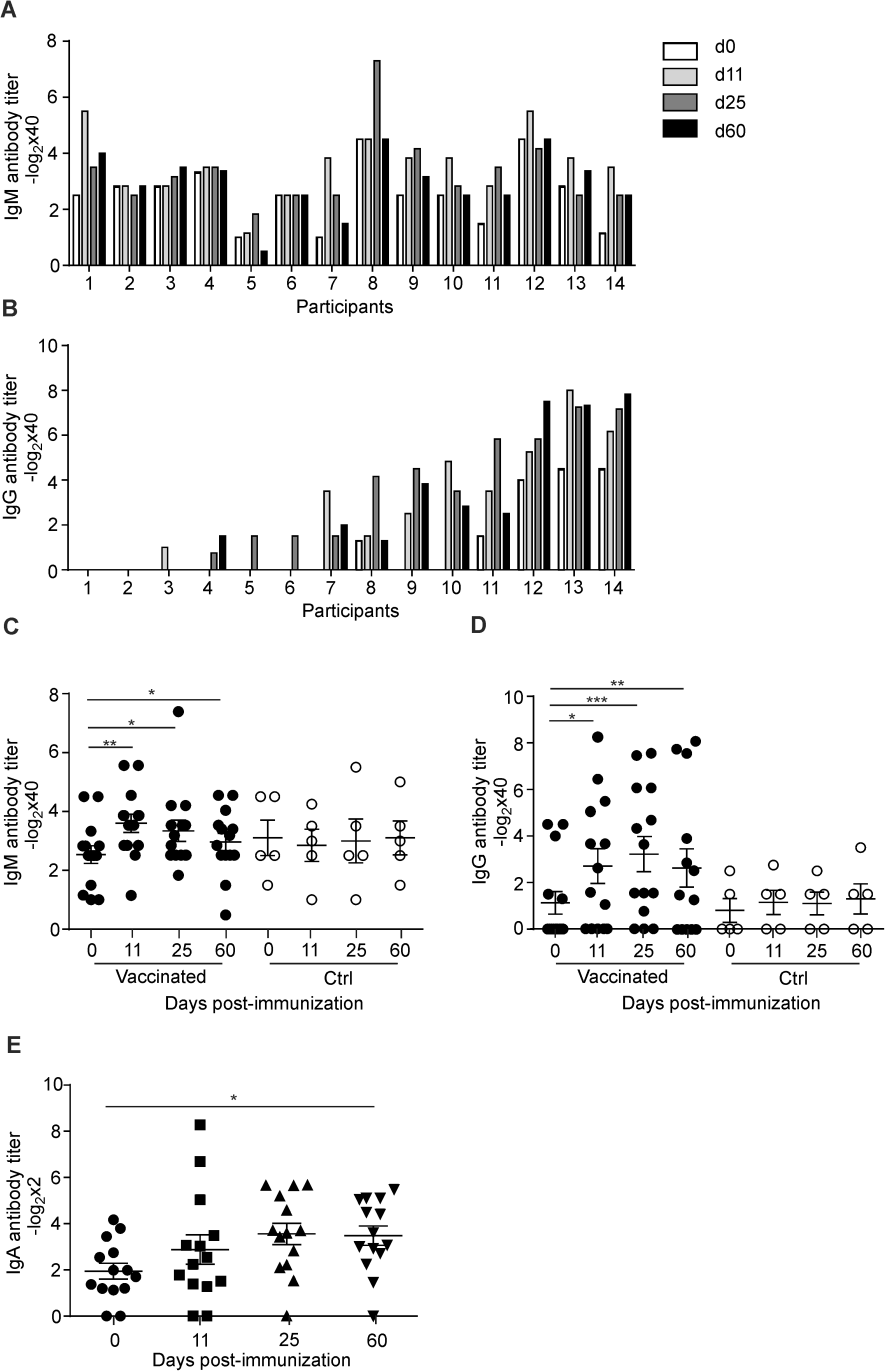
Induction of anti-OmpC/F-specific porin antibodies in serum and stool after Ty21a vaccination. (A) IgM and (B) IgG, OmpC/F-specific antibody titers in serum samples from vaccinated subjects were analyzed by ELISA at the indicated time points after vaccination; participant numbers assigned according to increasing IgG reactivity. (C and D) Kinetics of anti-OmpC/F IgM (C) and IgG (D) antibody titers in vaccinated or control (Ctrl) individuals. Dots represent individual values; bars indicate mean ± SEM. (E) *S*. Typhi OmpC/F-specific IgA was determined from stool samples from vaccinated or Ctrl subjects by ELISA. Dots represent individual values; bars represent mean ± SEM. Statistical analysis was performed using one way ANOVA with Dunnett’s multiple comparison test for comparisons between individuals of the same group at different time points (pre- versus post-vaccination) (*, P< 0.05; **, P< 0.01 ***, P<0.001).

Humoral responses against membrane proteins and O-9,12 antigen can be detected as early as 7 days after vaccination with Ty21a [6, 9], which is consistent with our finding that vaccinated individuals exhibited an increase of ASC in peripheral blood at day 11 post vaccination (Fig 2C). To assess whether the elevated B cell response was directed against *S*. Typhi porins OmpC and F, we determined anti-OmpC/F IgG and IgM antibody titers before and after vaccination. We found that all individuals showed IgM seroreactivity against OmpC and F and that exposure to the live attenuated vaccine did not drastically change the anti-OmpC/F IgM response (Fig 3A). In contrast, only 5 out of 14 individuals showed pre-existing anti-OmpC/F IgG antibodies and all but 2 individuals responded with an increase of specific IgG titers at one or more time points post vaccination (Fig 3B). Despite the high inter-individual variation, the pre- versus post-vaccination antibody titers were statistically significant at all time points for both anti-OmpC/F IgM (Fig 3C) and IgG (Fig 3D). OmpC/F-specific IgM antibody titers peaked at day 11 post vaccination (Fig 3C), while IgG values were highest at day 25 (Fig 3D). Likewise, mean anti-OmpC/F IgA antibodies in stool reached highest levels at day 25 and remained significantly elevated until day 60 post vaccination (Fig 3E). These data indicate that optimal antibody responses against *S*. Typhi OmpC and F develop over a period of two months.

### Polyfunctional CD4^+^ T cell responses after Ty21a vaccination

**Fig 4:**
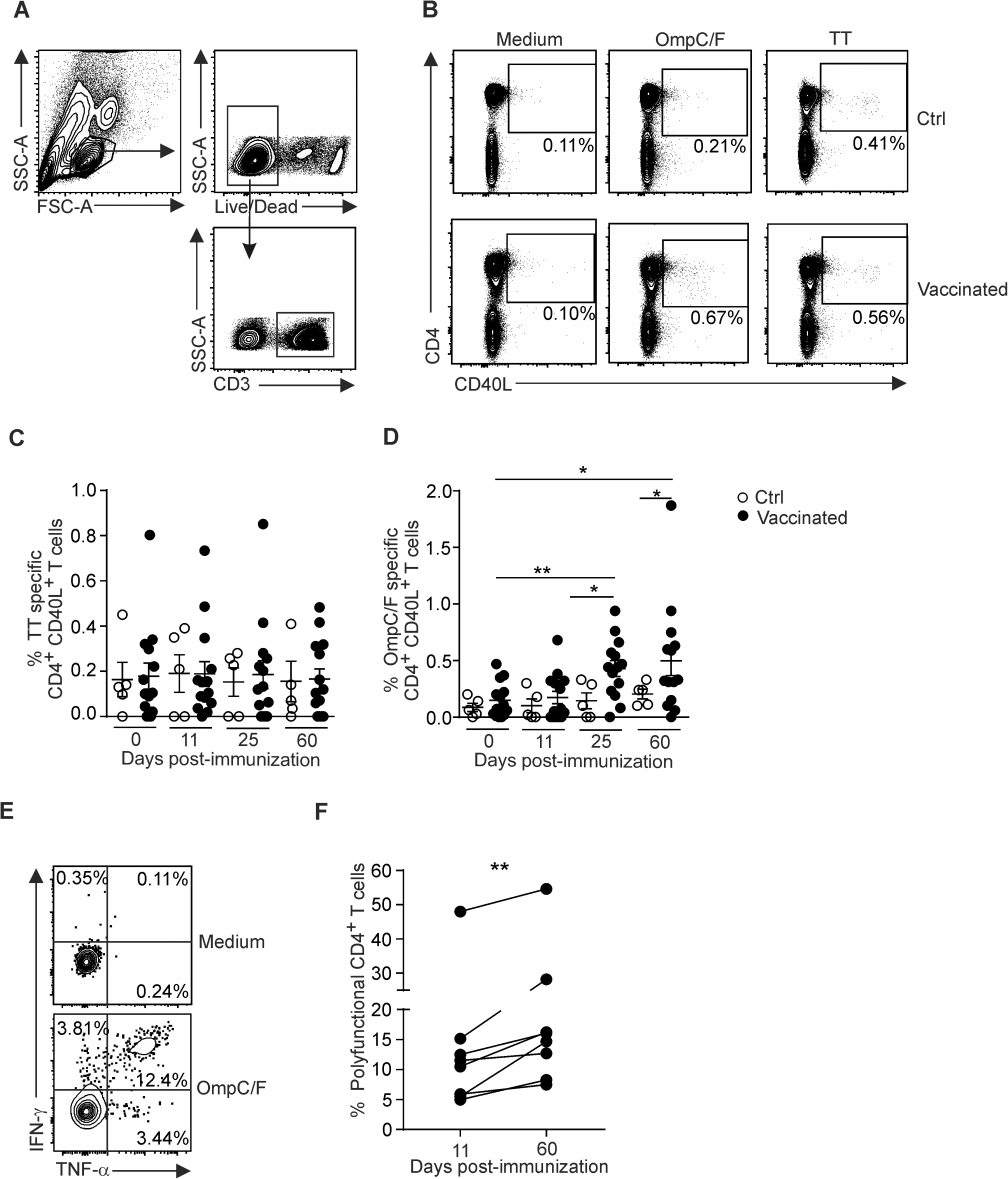
Activation profile of OmpC/F-specific CD4^+^ T cells after Ty21a vaccination. PBMCs were collected from vaccinated or control (Ctrl) subjects at the indicated time points. Activation of CD4^+^ T cells was assessed by flow cytometry after 24 h incubation with the indicated antigen. (A) Gating strategy for FACS analysis. (B) Representative dot plots showing the proportions of CD4^+^ CD40L^+^ T cells of Ctrl (upper panels) or vaccinated (lower panels) subjects after stimulation with *S*. Typhi OmpC/F porins or tetanus toxoid (TT). (C and D) Proportions of CD4^+^ CD40L^+^ T cells stimulated with TT (C) or OmpC/F (D) at the indicated time points. Dots represent individual values; bars represent mean ± SEM. (E) Representative dot plot showing IFN- γ and TNF-α production out of CD4^+^ CD40L^+^ cells after stimulation using medium or OmpC/F porins. (F) Proportions of OmpC/F-specific polyfunctional cells (IFN- γ ^+^ and/or TNF- α^+^) out of CD4^+^ CD40L^+^ T cells at indicated time points after vaccination in responder individuals. Statistical analysis in panels C and D was performed using one way ANOVA with Dunnett’s multiple comparison test for comparisons between individuals of the same group at different time points (pre- versus post-vaccination) or unpaired Student’s *t* test with Welch’s correction for comparison between Ctrl and vaccinated groups. Statistical analysis in panel F was performed using paired Student’s *t* test for comparison between individuals of the same group (Day 11 versus Day 60) (*, P< 0.05; **, P< 0.01).

Immunity against *S*. Typhi requires the concerted action of both B cell and T cells responses whereby provision of IFN- γ and other cytokines by CD4^+^ T cells is key for the control of the bacterial infection [1, 15]. To characterize the *S*. Typhi-induced T cell response in humans in more detail, we analyzed the activation profile of CD4^+^ T cells from PBMCs using *in vitro* stimulation with *S*. Typhi OmpC/F porins or tetanus toxoid (TT) as a control antigen. As shown in Fig 3A and 3B, intracellular CD40L expression served as activation marker to distinguish antigen-specific CD4^+^ T cells [26] (Fig 4A and 4B). While TT-specific CD4^+^ T cell activation was not altered during the course for Ty21a vaccination (Fig 4C), the fraction of OmpC/F-specific CD4^+^ T cells in peripheral blood had significantly increased by day 25 and was further augmented until day 60 (Fig 4D). Moreover, a substantial fraction of CD40L-expressing CD4^+^ T cells produced both IFN- γ and TNF following exposure to the antigen (Fig 4E). We observed a significant increase of such polyfunctional CD4^+^ T cells from day 11 to day 60 post vaccination in peripheral blood (Fig 4F) indicating that the live attenuated Ty21a exerts a persisting differentiation stimulus for OmpC/F-specific T cells. Evaluation of OmpC/F-specific revealed only very limited TNF-α and IFN- γ production by CD8^+^ T cells from 6 out of the 14 vaccinated volunteers (data not shown) suggesting that exposure to OmpC/F in the context of oral Ty21a immunization elicits mainly long lasting CD4^+^ T cell and antibody responses.

## Discussion

A better understanding of immunological processes occurring during *S*. Typhi infection and the re-definition of potential targets with protective properties are required to improve efficacy of the current typhoid vaccines. In this study, we have analyzed the evolution of B and T cell responses elicited by the *S*. Typhi porins OmpC and F during the course of infection with the live attenuated *S*. Typhi strain Ty21a. Previous studies have evaluated immune responses against *S*. Typhi antigens such the LPS [5, 9], the flagellar H-antigen [5, 12], or the oligosaccharide O-antigen [7, 12, 30]. We found that vaccination Ty21a elicited a substantial increase in ASC in the peripheral blood and that OmpC/F-specific antibodies significantly increased indicating that a strong polyclonal B cell response had been elicited against these antigens. Since patients in convalescence stages of typhoid fever show circulating IgM and IgG directed against OmpC and F [20, 31], it is most likely that *S*. Typhi porins contribute to protection against typhoid fever. Hence, our study confirms that porins represent highly immunogenic targets that can elicit significant IgA, IgM and IgG which have been shown to confer protection against *Salmonella* infection [19, 21, 32].

*S*. Typhi is an intracellular pathogen that can only be controlled if efficient T cell responses are induced [1]. While several studies have shown that *S*. Typhi infection or vaccination with Ty21a induce CD4^+^ and CD8^+^ T cell responses against several antigens, the magnitude and kinetics of CD4^+^ T cell responses against OmpC and F porins following Ty21a administration had not been assessed. Our study shows that the majority of the volunteers generated specific CD4^+^ T cell responses against OmpC and F porins following Ty21a administration indicating that these antigens contribute to the global CD4^+^ T cell response elicited by *S*. Typhi. Moreover, we found that volunteers vaccinated with Ty21a generated OmpC and F specific CD4^+^ T cells with a multifunctional cytokine production profile. These findings are in line with the finding that Ty21a vaccination elicits multifunctional CD4^+^ and CD8^+^ T cell responses against multiple antigens [10, 14]. Our results suggest that a substantial fraction of multifunctional CD4^+^ T cells recognize OmpC and F porins following vaccination with Ty21a and–most likely–during *S*. Typhi infection. Since porins are highly conserved proteins in *Salmonella* species [33], it is possible that vaccination with *S*. Typhi porins could elicit cross-protection against other *Salmonella* serovars. Indeed, cross-reactive T cell responses have been described in volunteers vaccinated with Ty21a [10, 14]. Moreover, exposure to Ty21a induces a strong IgA response against Omps from *S*. Typhi and *S*. Paratyphi B, but not against Omps from *S*. Paratyphi A [34]. Hence, further studies of cellular and humoral immune responses directed against well-characterized *Salmonella* antigens are warranted to further optimize the current vaccines and to develop approaches that facilitate the induction of effective immune responses that are directed against protective antigens.

## Supporting information

S1 Fig Identification of CD38+ antibody secreting cells in peripheral blood following Ty21a vaccination. PBMCs were collected from vaccinated or control (Ctrl) subjects at the indicated time points and analysis of different B cell populations was performed by flow cytometry. (A) Gating strategy for FACS analysis of IgD^-^ CD38^-^ B cells (blue) or CD38^+^ antibody secreting cells (ASC, red); representative dots plots on the right show the proportions of ASC (CD38^+^ CD71^+^) in a vaccinated individual at the indicated time points. Proportions ASC in vaccinated (B) and Ctrl (C) individuals at the indicated time points. Dots represent individual values; bars indicate mean ± SEM. Statistical analysis was performed using one way ANOVA with Dunnett’s multiple comparison test for comparisons between individuals of the same group at different time points (pre- versus post-vaccination) (*, P< 0.05).
(PDF)

S1 File Patient information in English. (DOCX)

S2 File Patient information in German. (DOCX)

S3 File Consort checklist. (DOC)

S4 File Data tables. (XLSX)

S1 Protocol (PDF)
