# Supplementary material for: Evolution of Salmonella Typhi outer membrane protein-specific T and B cell responses in humans following oral Ty21a vaccination: A randomized clinical trial
Source: PLoS One. 2017 Jun 1;12(6):e0178669. doi: 10.1371/journal.pone.0178669 (PMC5453566; doi:10.1371/journal.pone.0178669)
Supplement: S1 Fig — PBMCs were collected from vaccinated or control (Ctrl) subjects at the indicated time points and analysis of different B cell populations was performed by flow cytometry. (A) Gating strategy for FACS analysis of IgD- CD38- B cells (blue) or CD38+ antibody secreting cells (ASC, red); representative dots plots on the right show the proportions of ASC (CD38+ CD71+) in a vaccinated individual at the indicated time points. Proportions ASC in vaccinated (B) and Ctrl (C) individuals at the indicated time points. Dots represent individual values; bars indicate mean ± SEM. Statistical analysis was performed using one way ANOVA with Dunnett’s multiple comparison test for comparisons between individuals of the same group at different time points (pre- versus post-vaccination) (*, P< 0.05). (PDF) [file pone.0178669.s001.pdf]

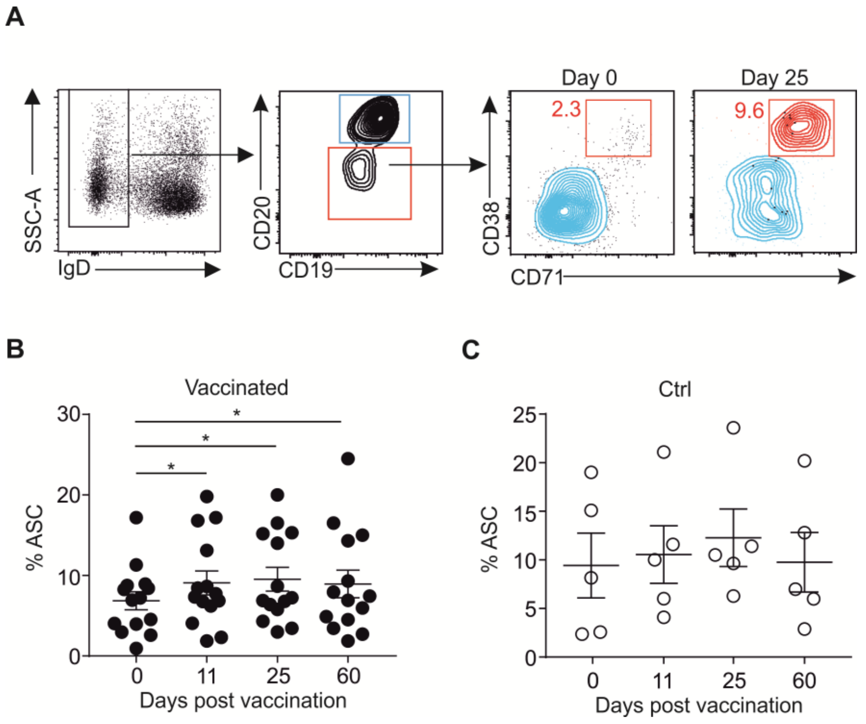

Supplementary Figure 1. Identification of CD38+ antibody secreting cells in peripheral blood following Ty21a vaccination. PBMCs were collected from vaccinated or control (Ctrl) subjects at the indicated time points and analysis of different B cell populations was performed by flow cytometry. (A) Gating strategy for FACS analysis of IgD- CD38- B cells (blue) or CD38+ antibody secreting cells (ASC, red); representative dots plots on the right show the proportions of ASC (CD38+ CD71+) in a vaccinated individual at the indicated time points. Proportions ASC in vaccinated (B) and Ctrl (C) individuals at the indicated time points. Dots represent individual values; bars indicate mean ± SEM. Statistical analysis was performed using one way ANOVA with Dunnett's multiple comparison test for comparisons between individuals of the same group at different time points (pre- versus post-vaccination) (*, P< 0.05).
