# Supplementary material for: Evolution of Salmonella Typhi outer membrane protein-specific T and B cell responses in humans following oral Ty21a vaccination: A randomized clinical trial
Source: PLoS One. 2017 Jun 1;12(6):e0178669. doi: 10.1371/journal.pone.0178669 (PMC5453566; doi:10.1371/journal.pone.0178669)
Supplement: S1 File — (DOCX) [file pone.0178669.s002.docx]

**Short version of participant information**

| Evaluation of specific immune responses against *Salmonella Typhi* porins after vaccination with the commercial live oral typhoid vaccine Ty21a Vivotif® (PORIMTIF) | **Details page** |
| --- | --- |
| **What we want to tell you:**  We would like to invite you to take part in our clinical study of vaccination against typhoid fever, in which we will investigate the immune responses induced by the commercial vaccine Vivotif. Vivotif can protect you against infection with *Salmonella Typhi*; a bacterium that causes Typhoid fever. | 3 |
| **What we hope to accomplish with our study:**  The aim of this study is to evaluate the immune responses induced after vaccination with the commercial live attenuated Salmonella vaccine Vivotif®; specifically the responses against a component of the vaccine that consists of proteins known as “Porins”. In addition, we would like to examine if the live bacteria contained in the vaccine formulation undergo genetic changes so called mutations after they are shed in your faeces. | 4 |
| **What participating in the study means for you:**  Prior to your enrolment into the study, you need to consent to participate. Thereafter it will be assessed, if you fulfil all criteria for enrolment. To ensure that your immune system will be able to respond adequately to the planned Vivotif vaccination, we will take a drop of blood from your finger (using a lancet) to evaluate if you are carrier of the Human Immunodeficiency Virus (HIV). HIV is known to weaken the immune system and therefore represents a risk during vaccination with Vivotif. In case the HIV test result is positive, you will be informed and you will not be allowed to participate in the study. We will offer you appropriate referral for further counselling and management.  If you are a woman, we will collect a urine sample to perform a pregnancy test during the first visit. You cannot participate in the study, if you are pregnant. During the entire study you need to use a reliable method of birth control.  After these assessments you may or may not receive the vaccine Vivotif. This will be decided based on randomization. This means that the selection of who will be placed in the Vivotif group or not is made randomly. In a situation like this, chance decides whether a participant receives the real medicinal product or not. 15 participants will be vaccinated and 5 participants will not be vaccinated. The probability that you will receive the test compound is 3 in 4.  Nevertheless all participants should attend to the study visits as indicated in this information sheet in order to accomplish the aim of this study.  During this study you will attend in total 7 visits at the Division of Infectious Diseases and Hospital Epidemiology, Kantonsspital St Gallen. The duration of the study from the first visit when you will receive the first dose of the vaccine until the last visit will be 60 days. The schedule of the visits is the following: visit 1 (Screening visit) 🡪 visit 2 (day 0) 🡪 visit 3 (day2) 🡪 visit 4 (day 4) 🡪 visit 5 (day 11) 🡪visit 6 (day 25) 🡪 visit 7 (day 60).  If you are in the Vivotif group, you take orally every two days the vaccine, a coated capsule. In total, you will receive 3 capsules.  During every visit a general health check-up will be done and your vital signs will be measured (body temperature, heart rate and blood pressure).  In visits 2-7 you will be asked to deliver a stool sample in a container that we will provide you. During visits 2, 5, 6 and 7 we will ask you to provide a blood sample, which will be obtained by venepuncture. | 4, 5 |
| **What are possible benefits or risks for you due to the study:**  Vaccination with Vivotif can protect you against Typhoid fever. The time of protection lasts approximately 3-5 years and therefore you may benefit from vaccination if you attend all study visits. However, not all recipients of Vivotif will be fully protected against typhoid fever and travellers should take all necessary precautions to avoid contact or ingestion of potentially contaminated food or water.  Vivotif is a safe vaccine; however for your own safety you should not take other specific drugs as indicated by your study doctor during vaccination. You should always follow the instructions of the medical personnel from the study. Adverse reactions are infrequent and mild, however some people may experience: diarrhea, abdominal pain, nausea, fever, headache, skin rash, vomiting, fatigue, malaise, chill, dizziness, having pin and needles, muscle and joint pain or urticaria in the trunk and/or extremities. Any serious adverse reactions related to the administration of the vaccine should be reported to your study doctor. | 6 |
| **What rights do you have if you participate in the study:**  Your participation in this study is voluntary. If you decide to participate, you can withdraw from the study at any time. You do not need to justify your decision. During the study, we will collect medical data about you. We will also collect samples of your faeces and blood. If you withdraw from the study at a later date, the samples collected so far will be used for the study purposes. | 6 |
| **What duties you have if you participate in the study:**  If you participate, you need to follow the instruction of your study doctor for your own safety. Importantly, you should avoid ingesting drugs, specified by the doctor during the study period. You should take all necessary precautions to avoid contact or ingestion of potentially contaminated food or water. | 6 |
| **What will happen with your data:**  We will comply with all data protection laws. We will use your data only for the purposes of the study. Everyone involved must maintain confidentiality. | 7 |
| **What you agree to when you give your consent:**  In addition to this short version, you will find extensive additional information on the following pages. Those pages form an integral part of the participant information. By signing the declaration of consent, you confirm that you accept the entire document. | 7 |
| **Who you can contact:**  Prof. Dr. Pietro Vernazza PD Dr. Werner Albrich  Chief, Infectious Diseases Senior Physician, Infectious Diseases  and Hospital of Epidemiology and Hospital of Epidemiology  Kantonsspital St. Gallen Kantonsspital St. Gallen  Rorschacher Strasse 95 Rorschacher Strasse 95  CH- 9007 St. Gallen CH- 9007 St. Gallen  Phone: +41 71 494 26 31 Phone: +41 71 494 26 53  Private CMN no. +41 79 666 2631 Private CMN no. +41 79 545 14 84 | 8 |

| **Long version of participant information** | | Page x of x |
| --- | --- | --- |
| 1 | Who can participate in the study | 3 of 9 |
| 2 | Objective of the study | 4 of 9 |
| 3 | General information on the study | 4 of 9 |
| 4 | Study structure for participants  (discontinuation of the study by the research team) | 4, 5 of 9 |
| 5 | Participants’ rights | 6 of 9 |
| 6 | Participants’ duties | 6 of 9 |
| 7 | Advantages for participants | 6 of 9 |
| 8 | Risks and stresses for participants | 6 of 9 |
| 9 | Optional: Other treatment options | 6 of 9 |
| 10 | Findings | 7 of 9 |
| 11 | Data confidentiality | 7 of 9 |
| 12 | Further use of material and data | 7 of 9 |
| 13 | Compensation for participants | 7 of 9 |
| 14 | Project financing | 7 of 9 |
| 15 | Contact | 8 of 9 |

*Project title in German*

Evaluation of specific immune responses against Salmonella Typhi porins after vaccination with the commercial live oral typhoid vaccine Ty21a Vivotif® (PORIMTIF)

Sponsor: Kantonsspital St Gallen (KSSG), Medizinische Departement I,

Infektiologie/Spitalhygiene, Prof. Dr. med. Pietro Vernazza

Dear Participant,

We are the staff at the Division of Infectious Diseases and Hospital Epidemiology, KSSG. We would like to invite you to take part in our clinical study of vaccination against typhoid fever, in which we will investigate the immune responses induced by the commercial vaccine Vivotif.

# **Who can participate in the study**

Any healthy volunteers between 18 and 50 years old who are: either employees at the Kantonalsspital St Gallen (except personnel from the Division of Infectious Disease/Hospital Epidemiology and the Institute of Immunobiology) or permanent residents in the Canton of St Gallen.

You cannot participate in case that you:

- Received an oral vaccine against Salmonella in the past 3 years
- Suffered from gastrointestinal infection caused by any Salmonella during the past 3 years
- Are infected with the Human Immunodeficiency Virus or have any known immune deficiency.
- Are pregnant or currently planning to get pregnant.
- Are not able to use at least one method of birth control (in women) during the course of the study.
- Received systemic corticosteroid treatment in the past 30 days
- Used antibiotics 1 week preceding and during the present study

# **Objective of the study**

The objective of this study is to evaluate the specific immune responses after vaccination with the commercial vaccine Vivotif® in healthy volunteers. This medicinal product has the following active ingredient: around 2 billion live germs of Viable *S. Typhi* Ty21a colony. These are germs with weakened virulence but closely related to pathogenic germs and are therefore activators of the immune system, but without the pathogenic effects.

# **General information on the study**

*Salmonella Typhi* is the etiological agent of typhoid fever, an acute, febrile enteric disease. Typhoid fever continues to be an important disease in many parts of the world. Travellers entering infected areas are at risk of contracting typhoid fever following the ingestion of contaminated food or water. Typhoid fever is considered to be endemic in most areas of Central and South America, the African continent, the Near East and the Middle East, Southeast Asia and the Indian subcontinent.

The majority of typhoid cases respond favourably to antibiotic therapy. However, the emergence of multi-drug resistant strains has greatly complicated therapy and cases of typhoid fever that are not diagnosed, treated late or treated with ineffective drugs can be fatal. Approximately 2–4% of acute typhoid cases result in the development of a chronic carrier state. These non-symptomatic carriers are the natural reservoir for *S. typhi* and can serve to maintain the disease in its endemic state or to directly infect individuals.

Virulent strains of *S. typhi* upon ingestion are able to pass through the acid barrier of the stomach, colonize the intestinal tract, penetrate the lumen and enter the lymphatic system and blood stream, thereby causing disease. The *S. typhi* Ty21a vaccine is indicated for immunization of adults and children greater than 6 years of age against disease caused by *Salmonella Typhi*. Selective immunization against typhoid fever is recommended for the following groups: 1) travellers to areas in which there is a recognized risk of exposure to *S. typhi*, 2) persons with intimate exposure (e.g. household contact) to a *S. typhi* carrier, and 3) microbiology laboratorians who work frequently with *S. typhi.* One capsule is to be swallowed approximately 1 hour before a meal with a cold or lukewarm water on alternate days, e.g., days 1, 3 and 5.

The duration of the study from the first visit when you will receive the first dose of the vaccine until the last visit will be 60 days. All the volunteers will be recruited at the Division of Infectious Diseases and Hospital Epidemiology, KSSG. In this randomised study, a total of 20 volunteers will be recruited: 15 will receive the vaccine (treated group) and 5 will not be vaccinated (control group) and you may be assigned by chance to either one or the other group. Regardless of the group in which you are assigned, the blood and stool samples from all the volunteers will be collected in the indicated visits.

This study will be carried out in accordance with Swiss laws and internationally recognised guidelines. It has been reviewed and endorsed by the competent cantonal ethics committee. You can also find a description of this study on the website of the Federal Office of Public Health: www.kofam.ch; www.humanforschunginfo.ch

# **Study structure for participants**

During this study you may receive the vaccine Vivotif, which can protect you against infection with *Salmonella Typhi*; a bacterium that causes Typhoid fever. Each dose of the vaccine consists of one coated capsule that is taken orally every two days. During this study you will attend during 7 visits at the Division of Infectious Diseases and Hospital Epidemiology, Kantonsspital St Gallen. In visits 2-7 you will be asked to deliver a stool sample in a container that we will provide you in your previous visit. Also, during four of the visits (number 2, 5, 6 and 7) we will ask you to provide a blood sample, which will be obtained by venepuncture.

To ensure that your immune system will be able to respond adequately to Vivotif vaccination, we will take a drop of blood from your finger (using a lancet) at visit 1 to evaluate, if you are carrier of the Human Immunodeficiency Virus (HIV), which is known to weaken the immune system and therefore represents a risk during vaccination with Vivotif. In case that the result of the test is positive, meaning that you are infected with the virus, you will be informed and you will not be allowed to participate in the study and we will offer you appropriate referral for further counselling and management. If you are female, during visit 1 we will collect a urine sample to perform a pregnancy test. Since the effects of the vaccine during pregnancy are not known, for safety reasons you will not be allowed to participate in the study, if you are found to be pregnant. If you are female, we will ask you to use at least one methods of birth control throughout the duration of the study.

During the study, some examinations will be related to the study and some examinations will be used to evaluate your health status either before or after the study starts. Examinations related to the study include: a) analysis of your blood to measure the amount of antibodies and cells of the immune system that are produced during vaccination and b) analysis of your faeces to measure the amount of antibodies and to evaluate how the vaccine is modified during transit through your gastrointestinal tract. Examinations not related to the study include: HIV test and pregnancy test (for females only).

During every visit a general health check-up will be done by qualified personnel from the Division of Infectious Diseases and Hospital Epidemiology, Kantonsspital St Gallen and your vital signs will be measured (body temperature, heart rate and blood pressure). The duration of the study from the 2th visit when you will receive the first dose of the vaccine until the last visit will be 60 days. The following chart summarizes the study plan to be followed:

| Study Periods | Screening | Vaccine administration (intervention period) | | | |  | |
| --- | --- | --- | --- | --- | --- | --- | --- |
| Visit**^1^** | 1 | 2 | 3 | 4 | 5 | 6 | 7 |
| Time (day) | -7 to -1 | 0 | 2 | 4 | 11 ± 1 | 25 ± 1 | 60 ± 1 |
| Patient Information and Informed Consent | X |  |  |  |  |  |  |
| Medical History | X |  |  |  |  |  |  |
| In- /Exclusion Criteria | X |  |  |  |  |  |  |
| Physical Examination | X |  |  |  |  |  |  |
| Vital Signs | X | X | X | X | X | X | X |
| Pregnancy Test | X |  |  |  |  |  |  |
| HIV-Test | X |  |  |  |  |  |  |
| Vivotif® vaccination**^2^** |  | 1^st^ dose | 2^th^ dose | 3^rd^ dose |  |  |  |
| Blood sample collection |  | X |  |  | X | X | X |
| Stool sample collection**^3^** |  | X | X | X | X | X | X |
| Serious adverse events |  | X | X | X | X | X | X |

**^1^**You will receive a phone call one day prior to each study visit as a reminder. **^2^**You will receive the vaccine in the study site during the indicated study visits. **^3^**You should deliver your faeces sample to the study site. We will provide you with sterile containers in the preceding study visit. *It is very important to obtain the stool sample during the same day of the study visit.* In case that you forget to bring the stool sample at the corresponding visit, the sample can be taken during the study visit.

We may have to prematurely eliminate you from the study. This may be necessary if there are ethical concerns, if the study cannot be completed because there are not enough patients, or if it needs to be stopped for other reasons or if there is concern about the safety of study participants: If this happens, we will offer to examine you one last time for your safety.

# **Participants’ rights**

You will only take part in this study if *you* want to be in it. No one is allowed to push you or talk you into it in any way. You do not need to justify your decision. If you do decide to participate, you are entitled to withdraw your consent at any time. You do not need to justify why you want to withdraw from the study either. You can ask any question you have about the study at any time. Please direct your questions to the person listed at the end of this document.

# **Participants’ duties**

If you take part in the study, you need to follow certain rules. These rules are designed to protect your safety and your health. We will do everything we can to support you. As a study participant, it is your duty to:

- Follow your investigator’s medical directions and respect the study plan. Avoid the use of the following medications during the clinical trial: corticosteroids, immune modulators, antimitotic drugs, proton-pump inhibitors, sulphonamides, antibiotics and anti-malaria drugs, such as mefloquine, chloroquine and proguanil. You should take all necessary precautions to avoid contact or ingestion of potentially contaminated food or water. Ask your doctor if you have any question about any medication you plan to take
- Keep your investigator informed about any changes in your health status: inform about any new symptoms, complaints or changes in your health.
- Inform your investigator about any treatment or therapy you are receiving from another physician and about any medicinal products you are taking. Please mention all medicinal products, even the ones you bought over the counter or without a prescription, including herbal teas, herbal remedies, etc. You also need to tell us about any alternative medicine products, such as homeopathy, or herbal medicines, etc.
- For women, use of at least one methods of birth control during the course of the study.

When we meet at the Division of Infectious Diseases and Hospital Epidemiology please bring along the stool sample in the containers provided in your last visit as indicated by the investigator.

# **Advantages for participants**

Taking part in this study might provide you with protection against Typhoid Fever. In addition, the results of the study could be significant for other people at risk of getting Typhoid Fever in the future.

# **Risks and burden for participants**

Vivotif is a safe vaccine; adverse reactions are infrequent and mild, nevertheless you may experience some adverse reaction(s) during vaccine administration included: diarrhea, abdominal pain, nausea, fever, headache, skin rash, vomiting, fatigue, malaise, chill, dizziness, having pin and needles, muscle and joint pain or urticaria in the trunk and/or extremities. Inform your investigator about any adverse event you may experience.

***For women who can become pregnant***

No data are yet available regarding the effects this medicinal product may have on a foetus. For that reason, female participants must use at least one method of contraception during the study (hormonal method [pill, coil] or a mechanical method such as a condom or a diaphragm.

Participants who become pregnant during the study must inform their investigator immediately and may no longer take part in the study. The investigator will discuss possible next steps with you. In such an event, you are requested to provide details on the progress and outcome of your pregnancy. Women who are breastfeeding may not take part in the study.

# **Other treatment options**

You do not have to participate in this study. The study treatment is a prophylactic treatment (to prevent getting a disease), not a therapeutic treatment (to treat a disease).

# **Findings**

The investigator will inform you during the study of all new findings that might influence the benefits of the study or your safety and thus your consent to take part in the study. You will receive this information orally and in writing.

# **Data confidentiality**

We will collect personal medical data about you for this study. These data will be encrypted. “Encryption” means that all identifying information (name, date of birth, etc.) is replaced by a code; persons unfamiliar with the code who do not have access to the encryption key will not be able to draw any conclusions as to your identity. Authorised and explicitly designated persons within the Infectious Diseases and Hospital Epidemiology will be able to access the data even without encryption. The encryption key will remain in the institution at all times.

The study may be reviewed while it is in progress. This review may be conducted by the authorities that assessed and approved the study before it began. The institution that pays for the study may also assess its progress. They are all working to ensure that the rules are followed and that your safety is not being compromised. The study supervisor may need to grant such third parties access to your personal and medical data for these reviews.

All persons who have anything to do with the study must observe the strictest confidentiality. We will not publish your name in any reports or publications, neither in print nor on the internet, nor in any other place.

There is a possibility that your health-related data and biological material will be sent to another biobank in Switzerland for analysis at a later date. That biobank must maintain the same standards as this one. The sponsor in Switzerland is responsible for complying with national and international data protection directives.

# **Further use of material and data**

You can withdraw from the study at any time if you wish to do so. We will still analyse the medical data and biological material (blood samples, tissue, etc.) that we have collected from you prior to that point in time, as not doing so would negatively impact the study as a whole.

After that, we will anonymise your data and your material, i.e. we will permanently delete the code that was previously assigned to you from those items. Then no one will be able to determine that the data and material belonged to you, besides your study physician.

# **Compensation for participants**

You will not be charged for participating in the study. Moreover, if you participate, you will receive an inconvenience compensation of 50 CHF per study visit at the end of the study, which includes transportation costs.

Under certain circumstances, the results of some medicinal product tests can contribute to the development of commercial products. By agreeing to take part in the study, you waive your entitlement to exploitation rights (especially patents) for the samples taken and the data obtained from them.

# **Project financing**

This study is funded in large part by the following foundations and/or organisations:

1. Gottfried und Julia Bangerter-Rhyner Foundation
2. Stanley Thomas Johnson Foundation
3. Division of Infectious Diseases and Hospital Epidemiology

# **Contact**

If anything is unclear or worrisome, or in case of emergencies that occur during or after the study, you may contact one of the persons below at any time.

PD Dr. Werner Albrich

Senior Physician

Infectious Diseases and Hospital Epidemiology

Kantonsspital St. Gallen

Rorschacher Strasse 95

CH- 9007 St. Gallen

Phone: +41 71 494 26 53

Private CMN no. +41 79 545 14 84

**Written Declaration of Consent for participation in a study**

- Please carefully read the entire form.
- If there is something you do not understand or would like to know, please ask.

| Study reference | Study Ref.  Ethikkommission des Kantons St. Gallen |
| --- | --- |
| Study title | Evaluation of specific immune responses against *Salmonella Typhi* porins after vaccination with the commercial live oral typhoid vaccine Ty21a Vivotif® (PORIMTIF)  *and lay German* |
| **Responsible institution (sponsor)** | Prof. Dr. Pietro Vernazza, Chief, Infectious Diseases and Hospital Epidemiology. Kantonsspital St. Gallen. Rorschacher Strasse 95. CH- 9007 St. Gallen. Phone: +41 71 494 26 31. Private CMN no. +41 79 666 2631. |
| Place of execution: | Division of Infectious Diseases and Hospital Epidemiology  Kantonsspital St. Gallen  Rorschacher Strasse 95  CH- 9007 St. Gallen |
| **Study supervisor**  Last name and first name (please print): | PD Dr. Albrich, Werner |
| **Participant**  Last name and first name (please print):  Date of birth: | Female  Male |

- The doctor whose signature appears below has informed me orally and in writing of the purpose and the structure of this study of Vivotif vaccination, of the expected effects and of possible advantages, disadvantages and potential risks.
- All of my questions about participating in the study have been answered to my satisfaction. I can keep the written participant information from 21.04.2015, Version 1.0 (two parts) and will receive a copy of my written declaration of consent. I accept the contents of the written participant information for the study named above.
- I am participating in this study of my own free will. I can withdraw my consent to take part at any time without explaining my reasons for doing so.
- I have been given sufficient time to reach a decision.
- I authorise you to inform my family physician about my participation in the study.

Yes 🞎 No 🞎

- I know that my personal data and biological material will be given to third parties for research purposes in encrypted form only. I authorise the study sponsor’s delegated experts, the authorities and the cantonal ethics committee to review my original data for auditing purposes, provided this is done in the strictest confidentiality.
- I am aware that I must fulfil certain duties while I am a participant in the study, as described in the participant information. For the sake of my health, the supervisor can eliminate me from the study at any time.

| Place, date | Signature of study participant |
| --- | --- |

**Confirmation by the investigator:** I hereby confirm that I have explained the substance, significance and implications of the study to this participant. I affirm that I will fulfil all of the obligations connected with this study in accordance with applicable law. If at any time during the study I am made aware of any aspects that could influence the participant’s willingness to take part in the study, I will inform him or her immediately.

| Place, date | Signature of investigator |
| --- | --- |
