# Supplementary material for: Evolution of Salmonella Typhi outer membrane protein-specific T and B cell responses in humans following oral Ty21a vaccination: A randomized clinical trial
Source: PLoS One. 2017 Jun 1;12(6):e0178669. doi: 10.1371/journal.pone.0178669 (PMC5453566; doi:10.1371/journal.pone.0178669)
Supplement: S2 File — (DOCX) [file pone.0178669.s003.docx]

**Kurzfassung der Studieninformation**

| PORIMTIF Studie:  Auswertung der spezifischen Immunantwort auf *Salmonella Typhi* Porine nach Schluckimpfung mit dem im Handel erhältlichen Lebendimpfstoff Vivotif® des Impfstammes S. Typhi Ty21a  Evaluation of specific immune responses against Salmonella Typhi porins after vaccination with the commercial live oral typhoid vaccine Ty21a Vivotif® | Details auf  Seite der Lang-fassung |
| --- | --- |
| **Was wir Ihnen mitteilen wollen:**  Wir möchten Sie hiermit bitten, an unserer klinischen Impfstudie gegen Abdominaltyphus teilzunehmen. Mit der Studie wollen wir die Immunantwort untersuchen, welche nach Einnahme der Schluckimpfung Vivotif^®^ hervorgerufen wird. Vivotif^®^ ist ein bereits zugelassener, im Handel erhältlicher Schluckimpfstoff zum Schutz gegen Abdominal-typhus, welcher durch das Bakterium *Salmonella Typhi* hervorgerufen, wird. Bei Vivotif® handelt es sich um einen sogenannten Lebendimpfstoff. Das bedeutet, dass dieser das Bakterium *Salmonella Typhi* in abgeänderter, nicht mehr schädlicher Form enthält. | 3 |
| **Was wir mit unserer Studie erreichen wollen:**  Durch Einnahme von Vivotif^®^ entwickelt der Körper eine Immunität gegen die Erreger des Abdominaltyphus. Das Ziel dieser Studie ist es, diese Immunantwort, nach Einnahme von Vivotif^®^ zu untersuchen. Speziell von Interesse ist die Immunantwort auf einen Bestandteil des Impfstoffes, welcher aus Eiweissen den „Porinen“ besteht. Zusätzlich möchten wir gerne untersuchen, ob die im Impfstoff enthaltenen lebenden Bakterien sich genetisch verändern, sogenannte Mutationen bilden, nachdem diese mit Ihrem Stuhl ausgeschieden werden. | 4 |
| **Was bedeutet die Teilnahme an der Studie für Sie:**  Bevor Sie an der Studie teilnehmen, müssen Sie zuerst schriftlich Ihre Einwilligung hierzu geben. Danach wird geprüft, ob Sie die Kriterien zur Studienteilnahme erfüllen. Um zu gewährleisten, dass Ihr Immunsystem im Stande ist angemessen auf die geplante Vivotif^®^ Impfung zu reagieren, werden wir Ihnen vom Finger einen Bluttropfen entnehmen (mit einer Lanzette) um zu erfahren, ob Sie Träger des humanen Immunschwäche-Virus (HIV) sind. Bekanntlich wird das Immunsystem durch HIV geschwächt, wodurch bei einer Impfung mit Vivotif^®^ ein Risiko besteht. Falls der HIV Test positiv ausfällt, werden wir Sie über das Resultat informiert. Sie können aber an der Studie nicht mehr teilnehmen. Wir werden Sie dann an die entsprechenden Beratungsstellen verweisen.  Falls Sie eine Frau sind, werden wir bei der ersten Visite, anhand einer Urinprobe, einen Schwangerschaftstest durchführen. Schwangere können an der Studie nicht teilnehmen. Während der ganzen Studiendauer müssen Sie eine zuverlässige Schwangerschaftsverhütung verwenden.  Nach diesen Abklärungen wird Ihnen die Vivotif^®^ Impfung verabreicht oder auch nicht. Dies wird anhand von Randomisierung entschieden. Das heisst, dass die Entscheidung, wer in die Vivotif^®^ Gruppe eingeteilt wird oder nicht rein zufällig ist. In solchen Situation entscheidet der Zufall, ob ein Teilnehmer das echte Medikament erhält oder nicht. In dieser Studie werden 15 Teilnehmer geimpft und 5 Teilnehmer werden nicht geimpft. Die Wahrscheinlichkeit, dass Sie das Medikament bekommen, beträgt somit 3 zu 1. Um das Studienziel zu erreichen sollten aber trotzdem alle Teilnehmer, wie in diesem Informationsblatt beschrieben, zu den vorgegebenen Visiten kommen.  Während der Studie haben Sie insgesamt 7 Visiten in der Abteilung für Infektiologie und Spitalhygiene am Kantonsspital St. Gallen. Die Studiendauer angefangen von der Visite bei der Sie die erste Impfdosis erhalten bis zur letzten Visite beträgt 60 Tage. Der Visitenplan ist wie folgt: Visite 1 (Screeningvisite) 🡪Visite 2 (Tag 0) 🡪Visite 3 (Tag 2) 🡪Visite 4 (Tag 4) 🡪Visite 5 (Tag 11) 🡪Visite 6 (Tag 25) 🡪Visite 7 (Tag 60).  Falls Sie in der Vivotif^®^ Gruppe sind, werden Sie jeden zweiten Tag eine orale Impfdosis in Form einer beschichteten Kapsel zu sich nehmen. Insgesamt werden Sie 3 Kapseln zu sich nehmen. Bei jeder Visite wird ein genereller Gesundheitscheck durchgeführt sowie Vitalparameter gemessen (Körpertemperatur, Herzfrequenz, Blutdruck). Für die Visiten 2-7 werden wir Sie bitten eine Stuhlprobe abzugeben. Dazu werden Ihnen spezielle Behälter zu Verfügung gestellt. Für die Visiten 2, 5, 6 und 7 werden wir mittels Venenpunktion jeweils eine Blutentnahme vornehmen. | 4, 5 |
| **Welcher Nutzen und welches Risiko mit der Studie für Sie verbunden sind:**  Eine Impfung mit Vivotif^®^ kann Sie gegen Abdominaltyphus schützen. Da der Impfschutz ungefähr 3-5 Jahre anhält, profitieren Sie eventuell von einer Impfung solange Sie alle vorgesehenen Impfdosen zu sich genommen haben. Dennoch kann es sein, dass nicht alle Teilnehmer, die Vivotif^®^ erhalten, einen vollständigen Schutz gegen Abdominaltyphus erhalten. Reisende sollten somit notwendige Vorsichtsmassnahmen treffen. Vivotif^®^ ist eine sichere Impfung. Sie sollten immer die Anweisungen des Studienpersonals befolgen. Unerwünschte Nebenwirkungen sind selten, trotzdem könnten folgende Symptome auftreten: Durchfall, Bauchschmerzen, Übelkeit, Fieber, Kopfschmerzen, Hautausschlag, Erbrechen, Müdigkeit, Unwohlsein, Schüttelfrost, Schwindel, Kribbeln, Muskel- und Gelenkschmerzen und allergische Reaktionen. Schwerwiegende unerwünschte Nebenwirkungen, welche auf die Einnahme der Impfung zurückzuführen sind, sollten Sie Ihrem Studienarzt melden. | 6 |
| **Welche Rechte Sie haben, wenn Sie an der Studie teilnehmen:**  Sie entscheiden frei, ob Sie an der Studie teilnehmen wollen oder nicht. Wenn Sie sich jetzt entscheiden teilzunehmen, können Sie jederzeit wieder aus der Studie aussteigen. Sie müssen Ihre Entscheidungen nicht begründen. Während der Studie erheben wir medizinische Daten über Sie. Ausserdem sammeln wir von Ihnen Blut und Stuhlproben. Wenn Sie später aus der Studie aussteigen, werden die bis dahin erhobenen Daten für die Studie verwendet. | 6 |
| **Welche Pflichten mit der Teilnahme an der Studie für Sie verbunden sind:**  Falls Sie an der Studie teilnehmen, müssen Sie, um Ihre eigene Sicherheit zu gewährleisten, die Anweisungen des Studienarztes befolgen. Wichtig ist, dass Sie während der Studie keine weiteren Medikamente einnehmen, welche nicht durch Ihren Studienarzt genehmigt wurden. Sie sollten alle notwendigen Vorsichtsmassnahmen treffen, um einen Kontakt oder die Aufnahme von eventuell verunreinigtem Wasser oder Nahrungsmitteln zu vermeiden | 6 |
| **Was mit Ihren Daten geschieht:**  Wir halten alle gesetzlichen Regeln des Datenschutzes ein. Wir verwenden Ihre Daten nur im Rahmen der Studie. Alle Beteiligten unterliegen der Schweigepflicht. | 7 |
| **Was Sie mit Ihrer Einwilligung bestätigen:**  Nebst dieser Kurzfassung finden Sie auf den nachfolgenden Seiten umfassende Zusatzinformationen. Diese sind wesentlicher Bestandteil der Studieninformation. Mit der Unterzeichnung der Einwilligungserklärung akzeptieren Sie das gesamte Dokument. | 7 |
| **An wen Sie sich wenden können:**  Prof. Dr. Pietro Vernazza PD Dr. Werner Albrich  Chefarzt, Infektiologie Oberarzt, Infektiologie  und Spitalhygiene und Spitalhygiene  Kantonsspital St. Gallen Kantonsspital St. Gallen  Rorschacher Strasse 95 Rorschacher Strasse 95  CH- 9007 St. Gallen CH- 9007 St. Gallen  Tel: +41 71 494 26 31 Tel +41 71 494 26 53  Private Handy Nr. +41 79 666 2631 Private Handy Nr. +41 79 545 14 84 | 8 |

**Langfassung der Studieninformation**

| **Inhaltsverzeichnis** | | Seite x von x |
| --- | --- | --- |
| 1 | Auswahl der Personen, die an der Studie teilnehmen können | 3 von 9 |
| 2 | Ziele der Studie | 4 von 9 |
| 3 | Allgemeine Informationen zur Studie | 4 von 9 |
| 4 | Ablauf für die Teilnehmenden  (Abbruch der Studie durch die Forschenden) | 4, 5 von 9 |
| 5 | Rechte der Teilnehmenden | 6 von 9 |
| 6 | Pflichten der Teilnehmenden | 6 von 9 |
| 7 | Nutzen für die Teilnehmenden | 6 von 9 |
| 8 | Risiken und Belastungen für die Teilnehmenden | 6 von 9 |
| 9 | Andere Behandlungsmöglichkeiten | 7 von 9 |
| 10 | Ergebnisse | 7 von 9 |
| 11 | Vertraulichkeit der Daten | 7 von 9 |
| 12 | Weitere Verwendung von Material und Daten | 7 von 9 |
| 13 | Entschädigung für Teilnehmende | 7 von 9 |
| 14 | Finanzierung der Studie | 8 von 9 |
| 15 | Kontaktperson(en) | 8 von 9 |

PORIMTIF Studie:

Auswertung der spezifischen Immunantwort auf *Salmonella Typhi* Porine nach Schluckimpfung mit dem im Handel erhältlichen Lebendimpfstoff Vivotif® des Impfstammes S. Typhi Ty21a

Evaluation of specific immune responses against Salmonella Typhi porins after vaccination with the commercial live oral typhoid vaccine Ty21a Vivotif®

Sponsor: Kantonsspital St Gallen (KSSG), Medizinische Departement I,

Infektiologie/Spitalhygiene, Prof. Dr. med. Pietro Vernazza

Sehr geehrte Dame, sehr geehrter Herr

Wir sind Mitarbeiter des Fachbereichs für Infektiologie und Spitalhygiene am KSSG. Wir möchten Sie einladen an unserer klinischen Impfstudie gegen Abdominaltyphus teilzunehmen. Die Studie untersucht die Immunantwort, welche nach Einnahme der bereits im Handel erhältlichen Schluckimpfung Vivotif^®^ hervorgerufen wird.

# **Auswahl der Personen, die an der Studie teilnehmen können**

Alle gesunden Personen zwischen 18 und 50 Jahren die nicht in der Abteilung der Infektiologie/Spitalhygiene oder am Institut für Immunbiologie des Kantonsspitals St Gallen angestellt sind.

Sie können an der Studie nicht teilnehmen falls Sie:

- Eine Salmonellen Schluckimpfung während den letzten 3 Jahren erhalten haben
- Eine Magen-Darm Infektion, aufgrund von Salmonellen, während den letzten 3 Jahren gehabt haben
- Mit dem humanen Immunschwäche-Virus (HIV) infiziert sind oder eine andere bekannte Immunschwäche haben
- Schwanger sind oder planen schwanger zu werden
- Während der Studiendauer nicht wenigstens ein Verhütungsmittel benutzen können (Frauen)
- Eine systemische Corticosteroid-Behandlung, während den letzten 30 Tagen, erhalten haben
- Ein Antibiotikum während der vorangegangen Woche sowie in der aktuellen Studie einnehmen

# **Ziele der Studie**

Das Ziel dieser Studie ist es die spezifisch hervorgerufene Immunantwort bei gesunden Personen, nach Einnahme der im Handel erhältlichen Schluckimpfung Vivotif^®^, zu untersuchen. Der Impfstoff enthält folgende aktive Inhaltsstoffe: ca. 2 Milliarden lebende Bakterien aus der *S. Typhi* Ty1a Kolonie. Dies sind Bakterien mit einer geschwächten Fähigkeit zur Ansteckung aber in enger Verwandtschaft mit der krankheitserregenden Bakterie, was somit zur Aktivierung des Immunsystems führt aber ohne deren krankheitserregenden Auswirkungen.

# **Allgemeine Informationen zur Studie**

Salmonellen sind weltweit die häufigsten bakteriellen Erreger gastrointestinaler Infektionen und stellen ein grosses Gesundheitsproblem in Entwicklungsländern aber auch in Industrienationen dar. Reisende die sich in Risikogebiete begeben, können sich nach Aufnahme von verunreinigtem Wasser oder Nahrungsmitteln mit Abdominaltyphus anstecken.

In den meisten Fällen sprechen die Patienten gut auf Antibiotika an. Trotzdem hat das Auftauchen von Erregerstämmen, die gegen verschiedene Medikamente unempfindlich sind, die Behandlung sehr erschwert. Nicht- oder zu spät diagnostizierter sowie falsch behandelter Abdominaltyphus kann tödlich sein. Ungefähr 2-4% akuter Typhusfälle können chronisch verlaufen und die Patienten somit zu sogenannten Dauerausscheidern werden. Dauerausscheider zeigen selbst keine Krankheitssymptome, sind aber Träger für *S. Typhi*, und können somit andere Personen anstecken.

Die Krankheit entsteht nach Einnahme ansteckender *S. Typhi* Stämme, die die Säurebarriere des Magens passieren, den Darmtrakt besiedeln, in die Hohlräume eindringen und somit in das lymphatische System sowie den Blutkreislauf gelangen. Die *S. Typhi* Ty21a ist ein Impfstoff gegen die durch *Salmonella Typhi* verursachte Krankheit und für Erwachsene und Kinder ab 5 Jahre geeignet. Eine selektive Schutzimpfung ist bei folgenden Gruppierungen empfohlen: 1) Reisende in Gebiete mit bekanntem *S. Typhi* Expositionsrisiko, 2) Personen welche intimen Kontakt zu *S. Typhi* Trägern haben (z.B. im gleichen Haushalt) und 3) Laboranten der Mikrobiologie die öfters mit *S. Typhi* Bakterien arbeiten.

Die Studiendauer angefangen von der Visite bei der Sie die erste Impfdosis erhalten, bis zur letzten Visite, beträgt 60 Tage. Die freiwillige Studienaufnahme findet in der Abteilung für Infektiologie und Spitalhygiene am Kantonsspital St. Gallen statt. In diese randomisierte Studie werden insgesamt 20 Freiwillige aufgenommen: 15 bekommen die Impfung (behandelte Gruppe) und 5 werden nicht geimpft (Kontrollgruppe). Randomisiert bedeutet, dass der Zufall bestimmt, ob Sie der einen oder anderen Gruppe zugeordnet werden, wie bei dem Werfen einer Münze. Ungeachtet welcher Gruppe Sie zugeteilt worden sind, werden von allen Studienteilnehmern zu den jeweiligen Visiten Blut und Stuhlproben gesammelt.

Wir machen diese Studie so, wie es die Gesetze in der Schweiz vorschreiben. Ausserdem beachten wir alle international anerkannten Richtlinien. Die zuständige Kantonale Ethikkommission hat die Studie geprüft und bewilligt. Eine Beschreibung dieser Studie finden Sie auch auf der Internetseite des Bundesamtes für Gesundheit: www.kofam.ch; [www.humanforschunginfo.ch](http://www.humanforschunginfo.ch)

# **Ablauf für die Teilnehmenden**

Während der Studie bekommen Sie eventuell die Vivotif^®^ Impfung, die Sie vor einer Infektion mit *Salmonella Typhi,* das Bakterium welches Thypus verursacht*,* schützen kann. Jede Impfdosis besteht aus einer beschichteten Kapsel die jeden zweiten Tag zu schlucken ist. Eine Kapsel wird jeweils nüchtern ungefähr 1 Stunde vor dem Essen mit kaltem oder lauwarmen Wasser (maximal 37°C) an den Tagen 1, 3 und 5 eingenommen.

Während der Studie werden Sie 7 Visiten, an der Abteilung für Infektiologie und Spitalhygiene am Kantonspital St. Gallen, wahrnehmen müssen. An den Visiten 2-7 werden Sie aufgefordert eine Stuhlprobe in einem Behälter, den wir Ihnen in einer vorherigen Visite aushändigen, abzuliefern. **Es ist sehr wichtig, dass Sie die Stuhlprobe an dem Tag nehmen, an dem Sie die Visite haben.** Zusätzlich wird während den Visiten 2, 5, 6 und 7 Blut mittels Venenpunktion entnommen. Um sicherzustellen, dass Ihr Immunsystem im Stande ist angemessen auf die Vivotif^®^ Impfung zu reagieren, werden wir Ihnen bei der ersten Visite vom Finger Bluttropfen entnehmen (mit einer Lanzette) um zu erfahren, ob Sie Träger des humanen Immunschwäche-Virus (HIV) sind. Bekanntlich wird das Immunsystem durch HIV geschwächt, wodurch bei einer Impfung mit Vivotif^®^ ein Risiko besteht. Falls der HIV Test positiv ausfällt, was bedeutet, dass Sie mit dem HIV Virus infiziert sind, werden wir Sie über das Resultat informieren. Sie können aber an der Studie nicht mehr teilnehmen. Wir werden Sie an die entsprechenden Beratungsstellen weiterverweisen. Falls Sie eine Frau sind, werden wir bei der ersten Visite, anhand einer Urinprobe, einen Schwangerschaftstest durchführen. Da die Auswirkung der Impfung während der Schwangerschaft nicht bekannt ist, können Sie aus Sicherheitsgründen an der Studie nicht teilnehmen, wenn Sie schwanger sind. Sind Sie eine Frau werden wir Sie auffordern, während der Studie mindestens 1 Verhütungsmittel zu benutzen

Folgende studienbezogene Untersuchungen werden durchgeführt: a) die Analyse Ihres Blutes um zu messen, wie viele Antikörper und Zellen Ihr Immunsystem während der Impfphase produziert und b) die Analyse Ihres Stuhls um zu messen wie viele Antikörper gebildet wurden und um zu untersuchen wie der Impfstoff verändert wird während dieser Ihren Magen-Darm-Trakt passiert. Folgende nicht-studienbezogene Untersuchungen werden durchgeführt: ein HIV Test und bei Frauen ein Urin-Schwangerschaftstest.

Während jedem Klinikbesuch wird durch einen Studienarzt der Abteilung für Infektiologie und Spitalhygiene am Kantonspital St. Gallen Ihr genereller Gesundheitszustand kontrolliert und Ihre Vitalzeichen (Körpertemperatur, Puls, und Blutdruck) werden gemessen.

Die folgende Tabelle fast den Ablauf zusammen:

| Untersuchung | Voruntersuchung | Impfphase | | | | Nachbeobachtung | |
| --- | --- | --- | --- | --- | --- | --- | --- |
| Visitennummer**^1^** | 1 | 2 | 3 | 4 | 5 | 6 | 7 |
| Zeit (Tag) | -7 bis -1 | 0 | 2 | 4 | 11 ± 1 | 25 ± 1 | 60 ± 1 |
| Studieninformation und schriftliches Einverständnis | X |  |  |  |  |  |  |
| Erfassung Ihrer Kranken-geschichte | X |  |  |  |  |  |  |
| Überprüfung der Studien-kriterien | X |  |  |  |  |  |  |
| Körperliche Untersuchung | X |  |  |  |  |  |  |
| Vitalzeichen | X | X | X | X | X | X | X |
| Urin-Schwangerschaftstest | X |  |  |  |  |  |  |
| HIV-Test | X |  |  |  |  |  |  |
| Vivotif^®^ Impfung**^2^** |  | 1. Dosis | 2. Dosis | 3. Dosis |  |  |  |
| Blutentnahme |  | X |  |  | X | X | X |
| Stuhlprobe**^3^** |  | X | X | X | X | X | X |
| Überprüfung auf Nebenwirkungen |  | X | X | X | X | X | X |

**^1^**Sie werden telefonisch einen Tag vor der jeweiligen Visite an den Termin erinnert. **^2^**Sie erhalten den Impfstoff in der Klinik während den Visiten. **^3^**Sie sollten Ihre Stuhlprobe in der Klinik abgeben. Wir werden Ihnen keimfreie Container bei der vorrangehenden Visite mitgeben. ***Es ist sehr wichtig, dass Sie die Stuhlprobe am gleichen Tag nehmen, an dem Sie die Visite haben.*** Falls Sie vergessen Ihre Stuhlprobe zur entsprechenden Visite mitzubringen, können Sie während der Visite eine Stuhlprobe abgeben.

Es kann sein, dass wir Sie von der Studie vorzeitig ausschliessen müssen. Das kann nötig sein, weil entweder ethische Bedenken bestehen, oder falls die Studie nicht beendet werden kann, da es nicht genügend Studienteilnehmer gibt, oder weil die Studie aus anderen Gründen beendet werden muss oder weil es Bedenken über die Sicherheit der Studienteilnehmer bestehen. Sollte dies eintreffen, bieten wir Ihnen an, Sie zur Sicherheit abschliessend zu untersuchen.

# **Rechte der Teilnehmenden**

Sie nehmen nur dann an dieser Studie teil, wenn Sie es wollen. Niemand darf Sie dazu in irgendeiner Weise drängen oder dazu überreden. Sie müssen nicht begründen, warum Sie nicht mitmachen wollen. Wenn Sie sich entscheiden mitzumachen, können sie diesen Entscheid jederzeit zurücknehmen. Sie müssen ebenfalls nicht begründen, wenn Sie aus der Studie aussteigen wollen.

Sie dürfen jederzeit alle Fragen zur Studie stellen. Wenden Sie sich dazu bitte an die Person, die am Ende dieser Studieninformation genannt ist.

# **Pflichten der Teilnehmenden**

Wenn Sie bei der Studie mitmachen, müssen Sie bestimmte Regeln beachten. Dies ist notwendig für Ihre Sicherheit und Gesundheit. Wir werden Sie dabei so gut wir können unterstützen. Als Studienteilnehmende/r sind Sie verpflichtet,

- den medizinischen Anweisungen Ihres Studienarztes zu folgen und sich an den Studienplan zu halten. Vermeiden Sie die Einnahme folgender Medikamente während der Studiendauer: Kortikosteroide, Arzneimittel die das Immunsystem beeinflussen, Arzneimittel zur Behandlung von Pilzkrankheiten, Arzneimittel zum Magenschutz, Sulfonsäureamide, Antibiotika und Anti-malaria Mittel, wie Mefloquin, Chloroquin und Proguanil. Sie müssen alle nötigen Vorsichtsmassnahmen treffen um Kontakt oder Aufnahme von eventuell verunreinigtem Essen und Wasser zu vermeiden. Wenn Sie zu Medikamenten die Sie einnehmen möchten Fragen haben, kontaktieren Sie vorher Ihren Studienarzt.
- Ihren Studienarzt über Änderungen in Ihrer Gesundheit zu informieren und neue Symptome, neue Beschwerden und Änderungen im Befinden zu melden
- Ihren Studienarzt über die gleichzeitige Behandlung und Therapien bei einem anderen Arzt und über die Einnahme von jeglichen Medikamenten zu informieren. Nennen Sie bitte alle Medikamente, auch solche, die Sie selbst gekauft haben, für die Sie kein Rezept brauchen, oder auch Kräutertees, pflanzliche Arzneien usw. Sie müssen uns auch Medikamente der Alternativmedizin nennen: Homöopathie, Spagyrik, usw.
- Frauen müssen mindestens eine Verhütungsmethode während der Dauer der Studie anwenden

Wenn Sie zu den Visiten in die Abteilung für Infektiologie und Spitalhygiene kommen, bringen Sie bitte Ihre Stuhlprobe in dem Behälter, den Ihr Studienarzt Ihnen in der vorangegangenen Visite aushändigt hat mit.

# **Nutzen für die Teilnehmenden**

Wenn Sie bei dieser Studie mitmachen, kann dies Sie eventuell gegen Typhus schützen. Ausserdem können die Resultate wichtig sein für andere, die das Risiko haben in der Zukunft Typhus zu bekommen.

# **Risiken und Belastungen für die Teilnehmenden**

Vivotif^®^ ist ein sicherer Impfstoff; Nebenwirkungen sind selten; trotzdem könnten folgende Nebenwirkungen während der Impfphase auftreten: Durchfall, Bauchschmerzen, Übelkeit, Fieber, Kopfschmerzen, Hautausschlag, Erbrechen, Müdigkeit, Unwohlsein, Schüttelfrost, Schwindel, Kribbeln, Muskel- und Gelenkschmerzen und allergische Reaktionen im Rumpf und den Gliedmassen. Informieren Sie Ihren Studienarzt über alle Nebenwirkungen, die Sie bemerken.

***Für Frauen, die schwanger werden können***

Es gibt noch keine Daten über die Wirkung des Medikaments auf den Fötus. Deshalb müssen Studienteilnehmerinnen während der Studie mindestens eine Verhütungsmethode (hormonale Methode [Pille, Spirale] oder eine mechanische Methode wie z.B. Diaphragma oder Präservativ für den Partner anwenden.

Teilnehmerinnen, die während der Studie schwanger werden, müssen ihren Studienarzt umgehend informieren und dürfen nicht weiter an der Studie teilnehmen. Der Studienarzt wird mit Ihnen das weitere Vorgehen besprechen. In diesem Fall werden Sie gebeten, Angaben über den Verlauf und den Ausgang der Schwangerschaft zu machen. Frauen, die stillen, sind von einer Studienteilnahme ausgeschlossen.

# **Andere Behandlungsmöglichkeiten**

Sie müssen bei dieser Studie nicht mitmachen. Die Studienbehandlung ist eine prophylaktische Behandlung (verhindern, dass Sie eine Erkrankung bekommen) und nicht eine therapeutische Behandlung (eine Erkrankung behandeln).

# **Ergebnisse aus der Studie**

Der Studienarzt wird Sie während der Studie über alle neuen Erkenntnisse informieren, die den Nutzen der Studie oder Ihre Sicherheit und somit Ihr Einwilligung zur Teilnahme an der Studie beeinflussen können. Sie werden die Information mündlich und schriftlich erhalten.

# **Vertraulichkeit der Daten**

Wir werden für diese Studie Ihre persönlichen und medizinischen Daten erfassen. Diese Daten werden wir verschlüsseln. Verschlüsselung bedeutet, dass alle Angaben, die Sie identifizieren könnten (z.B. Name, Geburtsdatum usw.), durch einen Code (Schlüssel) ersetzt sind, sodass für Personen, die den Code nicht kennen, keine Rückschlüsse auf Ihre Person möglich sind. Innerhalb der Abteilung für Infektiologie und Spitalhygiene am Kantonspital St. Gallen können die Daten durch berechtigte und klar bezeichnete Personen auch ohne Verschlüsselung eingesehen werden. Der Schlüssel bleibt immer in der Abteilung.

Es kann sein, dass die Studie während des Ablaufs überprüft wird. Dies können die Behörden tun, die sie vorab kontrolliert und bewilligt haben. Auch diejenige Institution, die die Studie bezahlt, kann den Ablauf überprüfen lassen. Sie alle sorgen dafür, dass die Regeln eingehalten werden und Ihre Sicherheit nicht gefährdet wird. Dazu muss der Leiter der Studie eventuell Ihre persönlichen und medizinischen Daten für solche Kontrollen offenlegen.

Alle Personen, die mit der Studie in irgendeiner Weise zu tun haben, müssen absolute Vertraulichkeit wahren. Wir werden Ihren Namen nirgends, in keinem Bericht, keiner Publikation, nicht gedruckt und nicht im Internet, veröffentlichen.

Es ist möglich, dass Ihre gesundheitsbezogene Daten und biologisches Material zu einem späteren Zeitpunkt an eine andere Biobank in der Schweiz zu Analysezwecken übersandt werden. Diese muss die gleichen Standards einhalten wie die vorliegende Biobank. Verantwortlich für die Einhaltung der nationalen und internationalen Richtlinien zum Datenschutz ist der Sponsor in der Schweiz.

# **Weitere Verwendung von Material und Daten**

Sie können jederzeit aus der Studie aussteigen, wenn Sie dies wünschen. Die medizinischen Daten, die wir bis dahin erhoben haben, und das biologische Material (Blutproben, Stuhlprobe etc.) von Ihnen werden wir trotzdem auswerten, weil sonst die ganze Studie ihren Wert verlieren würde.

Danach werden wir Ihre Daten und Ihr Material anonymisieren, d.h. wir werden endgültig Ihren code, der Ihren Proben zugeteilt wurde darauf löschen. Niemand wird danach mehr erfahren können, dass die Daten und das Material von Ihnen stammten.

# **Entschädigung für Teilnehmende**

Die Teilnahme an der Studie ist kostenfrei. Wenn Sie bei dieser Studie mitmachen, bekommen Sie dafür am Ende der Studie eine Entschädigung von 50.00 Fr. pro Studienvisite, welche auch Ihre Fahrkosten mit abdeckt.

Die Ergebnisse eines Medikamententests können unter Umständen dazu beitragen, kommerzielle Produkte zu entwickeln. Wenn Sie einwilligen mitzumachen, verzichten Sie damit zugleich auf die wirtschaftlichen Verwertungsrechte (insbesondere Patente) an den Ihnen entnommenen Proben und den daraus ermittelten Daten.

# **Finanzierung der Studie**

Diese Studie wird mehrheitlich von den folgenden Stiftungen und/oder Organisationen bezahlt:

1. Gottfried und Julia Bangerter-Rhyner Stiftung
2. Stanley Thomas Johnson Stiftung
3. Abteilung für Infektiolgie und Spitalhygiene des Kantonsspitals St. Gallen

# **Kontaktperson(en)**

Bei allen Unklarheiten, Befürchtungen oder Notfällen, die während der Studie oder danach auftreten, können Sie sich jederzeit an eine dieser Kontaktpersonen wenden.

Prof. Dr. Pietro Vernazza PD Dr. Werner Albrich

Chefarzt, Infektiologie Oberarzt, Infektiologie

und Spitalhygiene und Spitalhygiene

Kantonsspital St. Gallen Kantonsspital St. Gallen

Rorschacher Strasse 95 Rorschacher Strasse 95

CH- 9007 St. Gallen CH- 9007 St. Gallen

Tel: +41 71 494 26 31 Tel +41 71 494 26 53

Private Handy Nr. +41 79 666 2631 Private Handy Nr. +41 79 545 14 84**Schriftliche Einwilligungserklärung zur Teilnahme an einer Studie**

- Bitte lesen Sie dieses Formular sorgfältig durch.
- Bitte fragen Sie, wenn Sie etwas nicht verstehen oder wissen möchten.

| Nummer der Studie: (bei der zuständigen Ethikkommission des Kantons St. Gallen) |  |
| --- | --- |
| Titel der Studie: | Auswertung der spezifischen Immunantwort auf Salmonella Typhi Porine nach Schluckimpfung mit dem im Handel erhältlichen Lebendimpfstoff Vivotif® des Impfstammes S. Typhi Ty21a (PORIMTIF) |
| verantwortliche Institution (Sponsor): | Prof. Dr. Pietro Vernazza, Chefarzt, Infektiologie und Spitalhygiene, Kantonsspital St. Gallen, Rorschacher Strasse 95, CH- 9007 St. Gallen  Tel: +41 71 494 26 31, Private Handy Nr. +41 79 666 2631 |
| Ort der Durchführung: | Division of Infectious Diseases and Hospital Epidemiology  Kantonsspital St. Gallen  Rorschacher Strasse 95  CH- 9007 St. Gallen |
| Leiter der Studie:  Name und Vorname in Druckbuchstaben: | Prof. Dr. Pietro Vernazza |
| **Teilnehmerin/Teilnehmer:**  Name und Vorname in Druckbuchstaben:  Geburtsdatum: | weiblich  männlich |

- Ich wurde vom unterzeichnenden Arzt mündlich und schriftlich über den Zweck, den Ablauf der Studie mit dem Impfstoff Vivotif^®^ über die zu erwartenden Wirkungen, über mögliche Vor- und Nachteile sowie über eventuelle Risiken informiert.
- Meine Fragen im Zusammenhang mit der Teilnahme an dieser Studie sind mir zufriedenstellend beantwortet worden. Ich kann die schriftliche Studieninformation vom 08.05.2015, Version 1.0 (zwei Teile) behalten und erhalte eine Kopie meiner schriftlichen Einwilligungserklärung. Ich akzeptiere den Inhalt der zur oben genannten Studie abgegebenen schriftlichen Studieninformation.
- Ich nehme an dieser Studie freiwillig teil. Ich kann jederzeit und ohne Angabe von Gründen meine Zustimmung zur Teilnahme widerrufen, ohne dass ich deswegen Nachteile habe.
- Ich hatte genügend Zeit, meine Entscheidung zu treffen.
- Ich bin einverstanden, dass der Hausarzt über meine Teilnahme an der Studie informiert wird.

ja 🞎 nein 🞎

- Ich weiss, dass meine persönlichen Daten und Körpermaterialien nur in verschlüsselter Form zu Forschungszwecken weitergegeben werden können. Ich bin einverstanden, dass die zuständigen Fachleute des Auftraggebers der Studie, der Behörden und der Kantonalen Ethikkommission zu Prüf- und Kontrollzwecken in meine Originaldaten Einsicht nehmen dürfen, jedoch unter strikter Einhaltung der Vertraulichkeit.
- Ich bin mir bewusst, dass die in der Studieninformation genannten Pflichten während der Studie einzuhalten sind. Im Interesse meiner Gesundheit kann mich der Leiter jederzeit von der Studie ausschliessen.

| Ort, Datum | Unterschrift Studienteilnehmerin/Studienteilnehmer |
| --- | --- |

**Bestätigung des Studienarztes:** Hiermit bestätige ich, dass ich dieser Teilnehmerin/diesem Teilnehmer Wesen, Bedeutung und Tragweite der Studie erläutert habe. Ich versichere, alle im Zusammenhang mit dieser Studie stehenden Verpflichtungen gemäss dem geltenden Recht zu erfüllen. Sollte ich zu irgendeinem Zeitpunkt während der Durchführung der Studie von Aspekten erfahren, welche die Bereitschaft der Teilnehmerin/des Teilnehmers zur Teilnahme an der Studie beeinflussen könnten, werde ich sie/ihn umgehend darüber informieren.

| Ort, Datum | Name in Druckbuchstaben | Unterschrift Studienärztin/Studienarzt |
| --- | --- | --- |
