# Supplementary material for: Evolution of Salmonella Typhi outer membrane protein-specific T and B cell responses in humans following oral Ty21a vaccination: A randomized clinical trial
Source: PLoS One. 2017 Jun 1;12(6):e0178669. doi: 10.1371/journal.pone.0178669 (PMC5453566; doi:10.1371/journal.pone.0178669)
Supplement: S1 Protocol — (PDF) [file pone.0178669.s006.pdf]

Open, controlled monocentric clinical study to evaluate the specific immune responses against *Salmonella Typhi* porins after vaccination with the commercial live oral typhoid vaccine Ty21a Vivotif® (PORIMTIF)

Study Type:	Clinical trial with Investigational Medicinal Product (IMP)
Study Categorisation:	Risk category A
Study Registration:	Specific immune responses against *Salmonella Typhi* porins after vaccination with the commercial live oral typhoid vaccine Ty21a Vivotif®<br>Registration number: 14/039
Study Identifier:	PORIMTIF
Sponsor-Investigator:	KSSG, represented by Prof. Dr. Pietro Vernazza, Chief, Infectious Diseases and Hospital Epidemiology. Kantonsspital St. Gallen. Rorschacher Strasse 95. CH- 9007 St. Gallen. Phone: +41 71 494 26 31. Private CMN no. +41 79 666 2631.
Co-Investigator:	PD Dr. Werner Albrich. Senior Physician. Infectious Diseases and Hospital Epidemiology. Kantonsspital St. Gallen. Rorschacher Strasse 95. CH- 9007 St. Gallen. Phone: +41 71 494 26 53. Private CMN no. +41 79 545 14 84.
Investigational Product:	Typhoid Vaccine Live Oral Ty21a, Vivotif ®
Protocol Version and Date:	Protocol version 1.0, March16th, 2015.

CONFIDENTIAL

The information contained in this document is confidential and the property of the project leader. The information may not - in full or in part - be transmitted, reproduced, published, or disclosed to others than the applicable Competent Ethics Committee(s) and Regulatory Authority (ies) without prior written authorisation from the project leader except to the extent necessary to obtain informed consent from those who will participate in the study.

Signature Page(s)

Study number 14/039

Study Title Open, controlled monocentric clinical study to evaluate the specific immune responses against *Salmonella Typhi* porins after vaccination with the commercial live oral typhoid vaccine Ty21a Vivotif®

The Chairman, the Sponsor-Investigator, the Co-Investigator at study site and the trial statistician have approved the protocol version 1.0, dated March 16th, 2015 and confirm hereby to conduct the study according to the protocol, current version of the World Medical Association Declaration of Helsinki, ICH-GCP guidelines and the local legally applicable requirements.

Chairman:

Prof. Dr. Burkhard Ludewig
Head of Institute of Immunobiology
Kantonsspital St. Gallen
Rorschacher Strasse 95
CH- 9007 St. Gallen
Phone: +41-71-494 1090
Fax: +41-71-494 6321

__________________________ ____________________________________

Place/Date Signature

I have read and understood this trial protocol and agree to conduct the trial as set out in this study protocol, the current version of the World Medical Association Declaration of Helsinki, ICH-GCP guidelines and the local legally applicable requirements.

Sponsor-Investigator:

KSSG, represented by Prof. Dr. Pietro Vernazza,
Chief, Infectious Diseases and Hospital Epidemiology.
Kantonsspital St. Gallen. Rorschacher Strasse 95.
CH- 9007 St. Gallen.
Phone: +41 71 494 26 31.
Private CMN no. +41 79 666 2631.

Place/Date	Signature

I have read and understood this trial protocol and agree to conduct the trial as set out in this study protocol, the current version of the World Medical Association Declaration of Helsinki, ICH-GCP guidelines and the local legally applicable requirements.

Co-Investigator:

PD Dr. Werner Albrich
Senior Physician
Infectious Diseases and Hospital Epidemiology
Kantonsspital St. Gallen
Rorschacher Strasse 95
CH- 9007 St. Gallen
Phone: +41 71 494 26 53
Private CMN no. +41 79 545 14 84

Place/Date	Signature

I have read and understood this trial protocol and agree to conduct the trial as set out in this study protocol, the current version of the World Medical Association Declaration of Helsinki, ICH-GCP guidelines and the local legally applicable requirements.

Trial Statistician:

Rafael Sauter, MSc
Biostatistician
Clinical Trials Unit
Kantonsspital St Gallen
9007 St Gallen
Phone: + (0)71 494 36 74
Fax: + (0)71 494 65 19
Büro: Bedastrasse 1, Haus 89

______________________________ ______________________________________

Place/Date Signature

I have read and understood this trial protocol and agree to conduct the trial as set out in this study protocol, the current version of the World Medical Association Declaration of Helsinki, ICH-GCP guidelines and the local legally applicable requirements.

Table of Contents

STUDY SYNOPSIS 9

STUDY SUMMARY IN LOCAL LANGUAGE CAN BE PROVIDED HERE (GERMAN, FRENCH OR ITALIAN) 12

ABBREVIATIONS 13

STUDY SCHEDULE 14

1. STUDY ADMINISTRATIVE STRUCTURE 15

1.1 Sponsor 15

1.2 Chairman 15

1.3 Co-Investigator 16

1.4 Statistician 16

1.5 Laboratory 16

1.6 Monitoring institution 17

1.7 Data Safety Monitoring Committee 17

1.8 Any other relevant Committee, Person, Organisation, Institution 17

2. ETHICAL AND REGULATORY ASPECTS 18

2.1 Study registration 18

2.2 Categorisation of study 18

2.3 Competent Ethics Committee (CEC) 18

2.4 Competent Authorities (CA) 18

2.5 Ethical Conduct of the Study 18

2.6 Declaration of interest 18

2.7 Patient Information and Informed Consent 19

2.8 Participant privacy and confidentiality 19

2.9 Early termination of the study 19

2.10 Protocol amendments 19

3. BACKGROUND AND RATIONALE 20

3.1 Background and Rationale 20

3.2 Investigational Product (treatment, device) and Indication 21

3.3 Preclinical Evidence 23

3.4 Clinical Evidence to Date 23

3.5 Dose Rationale 24

3.6 Explanation for choice of comparator (or placebo) 24

3.7 Risks / Benefits 24

3.8 Justification of choice of study population 25

4. STUDY OBJECTIVES 26

4.1 Overall Objective 26

4.2 Primary Objective 26

4.3 Secondary Objectives 26

4.4 Safety Objectives 26

5. STUDY OUTCOMES 27

5.1 Primary Outcome 27

5.2 Secondary Outcomes 27

5.3 Other Outcomes of Interest 27

5.4 Safety Outcomes 27

6. STUDY DESIGN 28
6.1 General study design and justification of design 28
6.2 Methods of minimising bias 28
6.2.1 Randomisation 28
6.2.2 Blinding procedures 28
6.2.3 Other methods of minimising bias 28
6.3 Unblinding Procedures (Code break) 28
7. STUDY POPULATION 29
7.1 Eligibility criteria 29
7.2 Recruitment and screening 29
7.3 Assignment to study groups 30
7.4 Criteria for withdrawal / discontinuation of participants 30
8. STUDY INTERVENTION 31
8.1 Identity of Investigational Products (treatment) 31
8.1.1 Experimental Intervention (treatment) 31
8.1.2 Control Intervention (standard/routine/comparator treatment) 31
8.1.3 Packaging, Labelling and Supply (re-supply) 31
8.1.4 Storage Conditions 32
8.2 Administration of experimental and control interventions 32
8.2.1 Experimental Intervention 32
8.2.2 Control Intervention 32
8.3 Dose / Device modifications 32
8.4 Compliance with study intervention 32
8.5 Data Collection and Follow-up for withdrawn participants 33
8.6 Trial specific preventive measures 33
8.7 Concomitant Interventions (treatments) 33
8.8 Study Drug Accountability 34
8.9 Return or Destruction of Study Drug 34
9. STUDY ASSESSMENTS 35
9.1 Study flow chart(s) / table of study procedures and assessments 35
9.2 Assessments of outcomes 35
9.2.1 Assessment of primary outcome 35
9.2.2 Assessment of secondary outcomes 36
9.2.3 Assessment of other outcomes of interest 37
9.2.4 Assessment of safety outcomes 38
9.2.4.1 Serious Adverse Events 38
9.2.4.2 Vital signs 39
9.2.5 Assessments in participants who prematurely stop the study 39
9.3 Procedures at each visit 39
9.3.1 Visit 1. Screening visit, day -7 39
9.3.2 Visit 2. Intervention visit, day 0 39
9.3.3 Visit 3. Intervention visit, day 2 40
9.3.4 Visit 4. Intervention visit, day 4 40
9.3.5 Visit 5. Intervention visit, day 6 40
9.3.6 Visit 6. Follow-up visit, day 27 40
9.3.7 Visit 7. Follow-up visit, day 60 40

10. SAFETY **42**
10.1 Drug studies 42
10.1.1 Definition and assessment of serious adverse events and other safety related events 42
10.1.2 Reporting of serious adverse events (SAE) and other safety related events 43
10.1.3 Follow up of serious) Adverse Events 44
10.2 Medical Device Category C studies 44
10.2.1 Definition and Assessment of (Serious) Adverse Events and other safety related events 44
10.2.2 Reporting of (Serious) Adverse Events and other safety related events 44
10.2.3 Follow up of (Serious) Adverse Events 44
10.3 Medical Device Category A studies 44
10.3.1 Definition and Assessment of safety related events 44
10.3.2 Reporting of Safety related events 43
11. STATISTICAL METHODS **44**
11.1 Hypothesis 44
11.2 Determination of Sample Size 45
11.3 Statistical criteria of termination of trial 45
11.4 Planned Analyses 45
11.4.1 Datasets to be analysed, analysis populations 45
11.4.2 Primary Analysis 45
11.4.3 Secondary Analyses 45
11.4.4 Interim analyses 44
11.4.5 Safety analysis 45
11.4.6 Deviation(s) from the original statistical plan 46
11.5 Handling of missing data and drop-outs 46
12. QUALITY ASSURANCE AND CONTROL **47**
12.1 Data handling and record keeping / archiving 47
12.1.1 Case Report Forms 47
12.1.2 Specification of source documents 47
12.1.3 Record keeping / archiving 48
12.2 Data management 48
12.2.1 Data Management System 48
12.2.2 Data security, access and back-up 48
12.2.3 Analysis and archiving 48
12.2.4 Electronic and central data validation 48
12.3 Monitoring 48
12.4 Audits and Inspections 49
12.5 Confidentiality, Data Protection 49
12.6 Storage of biological material and related health data 49
13. PUBLICATION AND DISSEMINATION POLICY **50**
14. FUNDING AND SUPPORT **49**
14.1 Funding 49
14.2 Other Support 49
15. INSURANCE **49**
16. REFERENCES **50**

STUDY SYNOPSIS

**Sponsor-Investigator**	KSSG, represented by Prof. Dr. Pietro Vernazza
**Study Title:**	Open, controlled monocentric clinical study to evaluate the specific immune responses against *Salmonella Typhi* porins after vaccination with the commercial live oral typhoid vaccine Ty21a Vivotif®
**Short Title / Study ID:**	PORIMTIF
**Protocol Version and Date:**	Protocol version 1.0, March 16th, 2015
**Trial registration:**	Specific immune responses against *Salmonella Typhi* porins after vaccination with the commercial live oral typhoid vaccine Ty21a Vivotif®
**Study category and Rationale**	Risk category A. The medicinal product used in this trial is authorised in Switzerland and it will be used for the indication and with the dosage specified in the prescribing information.
**Clinical Phase:**	Clinical phase IV
**Background and Rationale:**	*Salmonella* species are the most common bacterial pathogens causing gastrointestinal infection worldwide and represent a considerable burden in both developing and developed countries. Although *Salmonella* is an intracellular pathogen, B cells play a crucial role in the control and generation of immunity against this bacterium, as shown in pre-clinical studies. Likewise, the main mechanism that confers protection after application of the current licensed vaccines in humans is mediated by antibodies.<br><br>Outer membrane proteins (Omps), also known as porins, represent important targets of the protective antibody response against *Salmonella* in humans. Highly purified *S. Typhi* porins OmpC and F induce long-lasting IgM and IgG bactericidal antibody responses in mice and exhibit intrinsic adjuvant activity. Notably, patients recovering from typhoid fever present both IgG and IgM circulating antibodies against porins and a porin-based vaccine candidate based *S. Typhi* porins has been tested in humans resulting to be safe and immunogenic following subcutaneous application. The induction of porin-specific immune responses after vaccination with live attenuated Salmonella vaccine Vivotif® has not been assessed.<br><br>Moreover, the specific antigenic targets that mediate protection during vaccination have not been identified. In this open, interventional trial, healthy volunteers will be vaccinated with Vivotif® commercial Salmonella vaccine following the standard immunization scheme (three capsules taken orally, one every other day). Blood and stool samples will be collected before the first oral dose and at days 7, 21 and 56 after the intake of the last oral dose and immune responses against *S. Typhi* porins will be assessed.
**Objective(s):**	The primary objective of this study is to evaluate the porin-specific immune responses after vaccination with the commercial live attenuated Salmonella vaccine Vivotif®.<br><br>The secondary objective is to evaluate the presence of bacteria that bear mutations in their DNA sequences in comparison with bacteria from the original inoculum administered during vaccination.

**Outcome(s):**	The primary endpoints of this study are:<br>Antibody levels of IgA, IgM and IgG specific against porins in serum and stool.<br>Number of porins-specific T cells from blood.<br>Number of porins-specific B cells in blood.<br>Secondary endpoints of this study are:<br>Bacteria from the original inoculum administered during vaccination bearing mutations in their DNA sequences.
**Study design:**	Open, interventional study, to evaluate the specific immune responses against *Salmonella Typhi* porins after vaccination with the commercial live attenuated *Salmonella* vaccine Vivotif®.
**Inclusion / Exclusion criteria:**	Healthy volunteers will be recruited among: a) employees at the Kantonalsspital St Gallen, excluding the personnel from the Division of Infectious Diseases and Hospital Epidemiology and Institute of Immunobiology and b) permanent residents in the Canton of St Gallen.<br><br>Inclusion criteria:<br>• Ability to understand the experimental nature of the vaccine evaluation and the participant informed consent form<br>• Written informed consent documented by date and signature to be obtained prior to any study specific procedure<br>• Age 18-50 years old<br>• Regular bowel movement (1+ defecation per day)<br>• Willingness to adhere to the strict timing schedule for the study evaluation<br>• Willingness to provide stool and blood samples in the indicated visits<br>Exclusion criteria:<br>• Previous use of an oral vaccine against *Salmonella* in the past three years<br>• Gastrointestinal infection caused by any *Salmonella* species during the past 3 years<br>• Positive HIV serology or any known immune deficiency<br>• Current or planned pregnancy during the course of the study<br>• Unwillingness to use at least one method of birth control in women of childbearing age during the course of the study<br>• Are breastfeeding<br>• Suffer from obstipation<br>• Suffer from hypersensitivity to any component of the vaccine or the enteric-coated capsule<br>• Use of an immune modulator in the past year<br>• Use of systemic corticosteroid treatment in the past 30 days<br>• Use of antibiotics within 1 week preceding and during the present study<br>• Current use of proton-pump inhibitors<br>• Participation in another study with investigational drug within the 30 days preceding and during the present study

**Measurements and procedures:**	Blood and stool samples will be collected before the first oral dose and at days 7, 21 and 56 after the intake of the last oral dose, anti-porins immune responses will be assessed: a) IgM and IgG levels in serum and stool determined by ELISA, b) Flow cytometry analysis of porins-specific T cells from blood and c) Analysis of porins-specific B cells in blood by ELISpot. Additionally, the presence of *Salmonella* genetic variants from the original inoculum administered will be analyzed. For this, bacterial culture from stool samples will be performed and the genome of *Salmonella* will be sequenced to analyse mutations in the original sequences of the bacteria populations.
**Study Product / Intervention:**	Vivotif® (Typhoid Vaccine Live Oral Ty21a) is a live attenuated vaccine for oral administration only. The vaccine contains the attenuated strain *Salmonella Typhi* Ty21a. One enteric-coated capsule of Vivotif® contains 2.0–10.0x10^9 colony-forming units (CFUs) of viable *S. Typhi* Ty21a and 5–50x10^9 CFUs of non-viable *S. Typhi* Ty21a bacterial cells.<br>3 doses of the vaccine are to be administered in alternate days (1, 3 and 5). One capsule is to be swallowed approximately 1 hour before a meal with a cold or lukewarm [temperature not to exceed body temperature, e.g., 37 °C (98.6 °F)] drink.
**Control Intervention (if applicable):**	The reference group (n=5) will not be vaccinated.
**Number of Participants with Rationale:**	15 participants in the vivotif ® vaccination group.<br>5 participants that will not be vaccinated.<br>Total: 20 participants.<br>This study is a pilot study; therefore, no sample size calculation was done.
**Study Duration:**	From the screening of the first participant to the end of follow up, the study will last approximately 3 months.
**Study Schedule:**	First participant-in: June, 2015.<br>Last participant-out: September, 2015.
**Investigator(s):**	PD Dr. Werner Albrich. Senior Physician. Division of Infectious Diseases and Hospital Epidemiology. Kantonsspital St. Gallen, Rorschacher Strasse 95, CH- 9007 St. Gallen. Phone: +41 71 494 26 53, Private CMN no. +41 79 545 14 84.<br>Prof. Dr. Burkhard Ludewig. Head of Institute of Immunobiology. Kantonsspital St. Gallen, Rorschacher Strasse 95, CH- 9007 St. Gallen. Phone: +41-71-494 1090, Fax: +41-71-494 6321.
**Study Centre(s):**	Single-centre. Kantonsspital St. Gallen, Rorschacher Strasse 95, CH- 9007 St. Gallen.
**Statistical Considerations:**	Differences to baseline will be investigated with a Wilcoxon signed rank test.
**GCP Statement:**	This study will be conducted in compliance with the protocol, the current version of the Declaration of Helsinki, the ICH-GCP (as far as applicable) as well as all national legal and regulatory requirements.

STUDY SUMMARY IN LOCAL LANGUAGE

Salmonellen sind weltweit die häufigsten bakterielllen Erreger gastrointestinaler Infektionen und stellen ein grosses Gesundheitsproblem in Entwicklungsländern aber auch in Industrienationen dar. Salmonella ist ein intrazellulärer Erreger. Um protektive Immunität zu erzielen braucht es dennoch neben der Salmonella-spezifischen T-Zellantwort auch B-Zellantworten (Antikörper).

In die äussere Membran eingelagerte Proteine, Outer membrane Proteins (Omps), die auch als Porine bezeichnet werden, sind wichtige Zielmoleküle bei der Immunantwort gegen Salmonella im Menschen. Nach überstandenem Typhus kann man im Serum sowohl IgG als auch IgM Antikörper gegen Porine finden. Ein porin-basierter Impfstoff (Porine von *Salmonella Typhi*) induzierte nach subkutaner Applikation sowohl T- als auch B-Zellantworten und erwies sich als sicher. Interessanterweise induzieren gereinigte *S. Typhi* Porine OmpC und OmpF eine lang anhaltende und bakterizide IgM- und IgG Antikörper-Antwort.

Die spezifischen Antigene, die mit den aktuell zugelassenen Impfungen den Schutz vor typhoidem Fieber vermitteln, sind bisher nicht bekannt. Ob eine Porin-spezifische Antwort nach Impfung mit dem attenuierten Salmonella-Impfstoff Vivotif® ausgelöst wird, wurde bisher nicht untersucht. In dieser offenen Interventionsstudie sollen gesunde Freiwillige mit Vivotif® gemäss Fachinformation immunisiert werden (oral, drei Kapseln an drei hintereinander folgenden Tagen). Blut- und Stuhlproben werden vor der Immunisierung und an den Tagen 7, 21 und 56 nach der Einnahme der letzten Dosis gesammelt und die Immunantworten gegen *S. Typhi* Porine werden gemessen.

Folgende Endpunkte werden untersucht: a) IgM und IgG Mengen im Serum und IgA im Stuhl gemessen mit ELISA. b) Durchflusszytometrische Quantifizierung Porin-spezifischer T- und B-Zellen aus Blut. c) Analyse Porin-spezifischer B-Zellen im Blut mittels ELISPOT. Zusätzlich wird analysiert, ob sich die genetische Grundinformation der attenuierten Impfbakterien ändert. Dazu werden Bakterienkulturen aus den Stuhlproben kultiviert und das Genom der Salmonellen wird sequenziert um evtl. auftretende Mutationen zu finden.

Zusammengefasst wird diese Studie aufklären, wie gut der attenuierte Impfstoff Ty21a Immunantworten gegen Porine in Gesunden induziert und wie genetisch stabil die attenuierten Bakterien sind. Dazu liegen bisher keine Informationen vor. Eine Anpassung der Bakterien im Verlauf der Infektion, könnte zu einer kontinuierlichen Evolution und auch zu einer Aufrechterhaltung der Krankheit führen. Die Untersuchungen erlauben die Identifizierung neuer therapeutischer Zielmoleküle für die Impfung gegen *S. Typhi*.

ABBREVIATIONS

AE	Adverse Event
CA	Competent Authority
CEC	Competent Ethics Committee
CRF	Case Report Form
ClinO	Ordinance on Clinical Trials in Human Research
eCRF	Electronic Case Report Form
CTCAE	Common terminology criteria for adverse events
GCP	Good Clinical Practice
IB	Investigator’s Brochure
Ho	Null hypothesis
H1	Alternative hypothesis
IMP	Investigational Medicinal Product
MD	Medical Device
SAE	Serious Adverse Events
SUSAR	Suspected Unexpected Serious Adverse Reaction
TMF	Trial Master File

STUDY SCHEDULE

Study Periods	Screening	Vaccine administration (intervention period)					
Visit[1]	1	2	3	4	5	6	7
Time (day)	-7 to -1	0	2	4	11 ± 1	25 ± 1	60 ± 1
Patient Information and Informed Consent	X						
Medical History	X						
In- /Exclusion Criteria	X						
Physical Examination	X						
Vital Signs	X	X	X	X	X	X	X
Pregnancy Test	X						
HIV-Test	X						
Vivotif® vaccination[2]		1st dose	2th dose	3rd dose			
Blood sample collection		X			X	X	X
Stool sample collection[3]		X	X	X	X	X	X
Serious adverse events		X	X	X	X	X	X

[1]Patients will receive a phone call one day prior to each study visit as a reminder. [2]Vaccine will be administered in the study site during the indicated study visits. [3]Stool samples will be received at the study site. Sterile containers are provided to the participants in the preceding study visit (i.e. sample collected at study visit number 2, will be obtained by the participant in a sterile container provided in study visit number 1). *It is very important to indicate to the patient that the stool sample should be obtained during the same day of the study visit and must be stored at 4°C until delivered to the personnel from the study.* In case that the participant forgets to bring the stool sample in the corresponding visit, the sample can be taken during the study visit.

1. STUDY ADMINISTRATIVE STRUCTURE

The clinical team from the Division of Infectious Diseases and Hospital Epidemiology, Kantonsspital St Gallen (KSSG) will have the following functions during the clinical study:

- Recruitment and enrolment of participants with adherence to inclusion and exclusion criteria.
- Registration of participants' information and obtaining Informed Consent.
- Elaboration and follow up of medical history of participants.
- Clinical evaluations including: physical examination, vital signs, pregnancy tests.
- Collection of blood and stool samples for evaluation of anti-porins immune responses.
- Application of Vivotif® vaccine.
- Registration and reporting of serious adverse events (SAE).

The clinical and administrative team from the Clinical Trials Unit (CTU), KSSG will evaluate the clinical protocol and all the documentation regarding the study. CTU personnel will perform a quality visit and ensure that the clinical trial is conducted according to clinical regulations.

Personnel from Institute of Immunobiology, KSSG will develop all immunological assays from blood and stool samples to evaluate anti-porins immune responses of study subjects. Samples will be taken during the study visits indicated in table 1 from section 9.1.

1.1 Sponsor-Investigator

KSSG, represented by Prof. Dr. Pietro Vernazza,

Chief, Infectious Diseases and Hospital Epidemiology.

Kantonsspital St. Gallen. Rorschacher Strasse 95.

CH- 9007 St. Gallen.

Phone: +41 71 494 26 31.

Private CMN no. +41 79 666 2631.

1.2 Chairman

Prof. Dr. Burkhard Ludewig

Head of Institute of Immunobiology

Kantonsspital St. Gallen, Rorschacher Strasse 95

CH- 9007 St. Gallen

Phone: +41-71-494 1090, Fax: +41-71-494 6321

Role in the study design: Collection, management, analysis and interpretation of data, writing the report and publication of the results.

1.3 Co-Investigator

PD Dr. Werner Albrich

Senior Physician

Division of Infectious Diseases and Hospital Epidemiology Kantonsspital St. Gallen

Rorschacher Strasse 95, CH- 9007 St. Gallen

Phone: +41 71 494 26 53, Private CMN no. +41 79 545 14 84

Role in the study: Recruitment and enrolment of participants, obtaining history, physical examination and Informed Consent, application of Vivotif® vaccine, reporting of serious adverse events, interpretation of data, writing the report and publication of the results.

1.4 Statistician ("Biostatistician")

Rafael Sauter, MSc

Biostatistician

Clinical Trials Unit

Kantonsspital St Gallen

9007 St Gallen

Phone: + (0)71 494 36 74

Fax: + (0)71 494 65 19

Büro: Bedastrasse 1, Haus 89

1.5 Laboratory

Two laboratories will be involved in the clinical trial. The *Institute of Immunobiology* will evaluate anti-porins immune responses of study subjects and the *Institute of Microbiology, ETH Zurich,* will perform the genetic analyses of *Salmonella* in stool samples.

Institute of Immunobiology

Medical Research Center (MFZ)

Kantonal Hosptial St. Gallen

Rorschacherstrasse 95

9007 St. Gallen, Switzerland

ETH Zürich

Institute of Microbiology

HCI G 417

Vladimir-Prelog-Weg 1-5/10

8093 Zurich, Switzerland

1.6 Monitoring institution

Clinical Trials Unit

Kantonsspital St Gallen

Bedastrasse 1

9000 St.Gallen, Switzerland

Telefon +41 71 494 35 12

Telefax +41 71 494 65 19

1.7 Data Safety Monitoring Committee

The risk category of the present study is A. Safety of Vivotif® live attenuated vaccine has been demonstrated in several clinical studies (please refer to section 3.4). Therefore, a data safety monitoring committee is not required for this study. A quality visit will be performed during the course of the study to ensure the adequate conduct of the clinical trial.

1.8 Any other relevant Committee, Person, Organisation, Institution

Not applicable.

2. ETHICAL AND REGULATORY ASPECTS

The decision of the Competent Ethics Committee (CEC) concerning the conduct of the study will be made in writing to the Sponsor-Investigator before commencement of this study. The clinical study can only begin once approval from all required authorities has been received. Any additional requirements imposed by the authorities shall be implemented.

2.1 Study registration

The study will be registered at the ISRCTN study database, which is listed in the WHO International Clinical Trials Registry Platform (ICTRP, http://www.who.int/ictrp/en/), and in the Swiss Federal Complementary Database (Portal).

2.2 Categorisation of study

Risk category A. The medicinal product used in this trial is authorised in Switzerland and it will be used for the indication and with the dosage specified in the prescribing information.

2.3 Competent Ethics Committee (CEC)

The responsible investigator at the investigation site will ensure the approval of the clinical study by an appropriately constituted CEC. Changes in the research activity and all unanticipated problems involving risks to humans; including planned or premature study end and the final report, will not be made to the protocol without prior Sponsor and CEC approval, except when is necessary to eliminate apparent immediate hazards to study participants.

Premature study end or interruption of the study is reported within 15 days. The regular end of the study is reported to the CEC within 90 days, the final study report shall be submitted within one year after study end.

2.4 Competent Authorities (CA)

The risk category of the study is A. Therefore CA approval is not necessary and will not be obtained for this study.

2.5 Ethical Conduct of the Study

The study will be carried out in accordance to the protocol and with principles enunciated in the current version of the Declaration of Helsinki, the guidelines of Good Clinical Practice (GCP) issued by ICH, the Swiss Law and Swiss regulatory authority's requirements. Further, no annual safety reports and information about stop/end dates of the study to Swiss regulatory authorities is required.

2.6 Declaration of interest

The sponsor-investigator and the Co-Investigator declare no conflicts of interest regarding independence, intellectual, financial and proprietary matters.

2.7 Patient Information and Informed Consent

The investigators will explain to each participant the nature of the study, its purpose, the procedures involved, the expected duration, the potential risks and benefits and any discomfort it may entail. Each participant will be informed that the participation in the study is voluntary and that he/she may withdraw from the study at any time and that withdrawal of consent will not affect his/her subsequent medical assistance and treatment.

The participant will be informed that his/her medical records may be examined by authorised individuals other than their treating physician.

All participants for the study will be provided a participant information sheet and a consent form describing the study and providing sufficient information for participant to make an informed decision about their participation in the study. Enough time needs to be given to the participant to decide whether to participate or not.

The participant information sheet and the consent form will be submitted to the CEC to be reviewed and approved. The formal consent of a participant, using the approved consent form, must be obtained before the participant is submitted to any study procedure.

The participant should read and consider the statement before signing and dating the informed consent form, and should be given a copy of the signed document. The consent form must also be signed and dated by the investigator (or his designee) and it will be retained as part of the study records.

2.8 Participant privacy and confidentiality

The investigator affirms and upholds the principle of the participant's right to privacy and that they shall comply with applicable privacy laws. Especially, anonymity of the participants shall be guaranteed when presenting the data at scientific meetings or publishing them in scientific journals.

Individual subject medical information obtained as a result of this study is considered confidential and disclosure to third parties is prohibited. Subject confidentiality will be further ensured by utilising subject identification code numbers to correspond to treatment data in the computer files.

For data verification purposes, authorised representatives of the Sponsor (-Investigator) and ethics committee may require direct access to parts of the medical records relevant to the study, including participants' medical history.

2.9 Early termination of the study

The Sponsor-Investigator may terminate the study prematurely in certain circumstances:

- Ethical concerns
- Insufficient participant recruitment
- When the safety of the participants is doubtful or at risk, respectively
- Alterations in accepted clinical practice that make the continuation of a clinical trial unwise
- Early evidence of benefit or harm of the experimental intervention

2.10 Protocol amendments

Substantial amendments are only implemented after approval of the CEC.

Under emergency circumstances, deviations from the protocol to protect the rights, safety and well-being of human subjects may proceed without prior approval of the sponsor and the CEC. Such deviations shall be documented and reported to the sponsor and the CEC as soon as possible.

3. BACKGROUND AND RATIONALE

3.1 Background and Rationale

Salmonella species are the most common bacterial pathogens causing gastrointestinal infection worldwide (1) and represent a considerable burden in both developing and developed countries (2). A recent estimation reports around 93.8 million cases of gastroenteritis due to *Salmonella* species and 155,000 deaths globally each year (2). Particularly typhoid fever and paratyphoid fever continue to be important causes of illness and death among children and adolescents in south-central and southeast Asia (3). Two typhoid vaccines are commercially available, Vivotif®, which consist of live attenuated *Salmonella Typhi* (Ty21a) for oral administration and ViCPS, which consist of Vi capsular polysaccharide administered parenterally. One concern about Ty21a vaccine is whether it reverts to virulence, but this has not been documented in past clinical trials (4).

Although *Salmonella* is an intracellular pathogen, B cells play a crucial role in the control and generation of immunity against this bacterium, as shown in pre-clinical studies (5). Likewise, the main mechanism that confers protection after application of the current licensed vaccines in humans is mediated by antibodies (6,7). Outer membrane proteins (Omps), also known as porins, represent important targets of the protective antibody response against *Salmonella* in humans, as patients recovering from typhoid fever present both IgG and IgM circulating antibodies against porins (8,9). During non typhoid salmonella (NTS) bacteremia, anti-*Salmonella* porins antibody responses have been shown to be critical in HIV-infected adults (10). Highly purified *S. Typhi* porins OmpC and F induce long-lasting IgM and IgG bactericidal antibody responses in mice (11) and exhibit intrinsic adjuvant activity (12). Moreover, a porins-based vaccine candidate based on *S. Typhi* porins OmpC and OmpF has been tested in humans and this vaccine candidate was found to be safe and immunogenic following subcutaneous application (9).

As mentioned, the main mechanism that confers protection after application of the current licensed vaccines in humans is mediated by antibodies. Nonetheless, the specific antigenic targets that mediate protection have not been clearly defined and the induction of porins-specific immune responses after vaccination with live attenuated *Salmonella* vaccine Vivotif® has not been assessed.

In this open, randomised, interventional trial, healthy volunteers will be vaccinated with Vivotif® commercial *Salmonella* vaccine following the standard immunization scheme (three capsules taken orally, one every other day). Blood and stool samples will be collected before the first oral dose and at days 7, 21 and 56 after the intake of the last oral dose. Specific immune responses against *S. Typhi* porins will be assessed: a) IgM and IgG levels in serum and IgA in stool determined by ELISA, b) Flow cytometry analysis of porins-specific T and B cells from blood and c) Analysis of porins-specific B cells in blood by ELISPot. Additionally, the presence of mutants from the original inoculum administered will be analysed. For this, bacterial culture from stool samples will be performed and the genome of *Salmonella* will be sequenced to analyse mutations in the original sequences of the bacteria populations.

Altogether, this study not only will provide information about the immune responses elicited against *S. Typhi* porins during vaccination of healthy subjects with the commercial live attenuated vaccine Ty21a against *Salmonella Typhi*, but also will allow to characterize the behaviour of the bacterial populations of a live attenuated vaccine in humans. These studies are important due to the lack of information regarding the molecular changes that the bacteria experience during the infection in humans that allow their adaptation to the host and the continuous evolution and perpetuation of the disease.

As a final remark, Vivotif® has been shown to be safe; nevertheless some precautions will be taken in consideration during the study. The vaccine will not be administered during an acute gastrointestinal illness or in individuals receiving sulfonamides and antibiotics since these agents may be active against the vaccine strain and prevent a sufficient degree of multiplication to occur in order to induce a protective immune response. If persistent diarrhea or vomiting is occurring, administration will be stopped.

3.2 Investigational Product (treatment, device) and Indication

Vivotif®
Typhoid Vaccine Live Oral Ty21a

Description

Vivotif® (Typhoid Vaccine Live Oral Ty21a) is a live attenuated vaccine for oral administration only. The vaccine contains the attenuated strain *Salmonella Typhi* Ty21a (13,14). Vivotif® is manufactured by PaxVax Berna. The vaccine strain is grown in fermenters under controlled conditions in medium containing a digest of yeast extract, an acid digest of casein, dextrose and galactose. The bacteria are collected by centrifugation, mixed with a stabilizer containing sucrose, ascorbic acid and amino acids, and lyophilized. The lyophilized bacteria are mixed with lactose and magnesium stearate and filled into gelatin capsules which are coated with an organic solution to render them resistant to dissolution in stomach acid. The enteric-coated, salmon/white capsules are then packaged in 3-capsule blisters for distribution. The contents of each enteric-coated capsule are shown in Table 1.

Table 1: Contents of one enteric-coated capsule of Vivotif® (Typhoid Vaccine Live Oral Ty21a)
Viable *S. Typhi* Ty21a 2.0–10.0x10^9 colony-forming units*
Non-viable *S. Typhi* Ty21a 5–50x10^9 bacterial cells
Sucrose 3.3 – 34.2 mg
Ascorbic acid 0.2 – 2.4 mg
Amino acid mixture 0.3 – 3.0 mg
Lactose up to 180 - 200 mg
Magnesium stearate 3.6–4.0 mg
*Vaccine potency (viable cell counts per capsule) is determined by inoculation of agar plates with appropriate dilutions of the vaccine suspended in physiological saline.

Clinical Pharmacology

Salmonella Typhi is the etiological agent of typhoid fever, an acute, febrile enteric disease. Typhoid fever continues to be an important disease in many parts of the world. Travellers entering infected areas are at risk of contracting typhoid fever following the ingestion of contaminated food or water. Typhoid fever is considered to be endemic in most areas of Central and South America, the African continent, the Near East and the Middle East, Southeast Asia and the Indian subcontinent (15).

The majority of typhoid cases respond favourably to antibiotic therapy. However, the emergence of multi-drug resistant strains has greatly complicated therapy and cases of typhoid fever that are not diagnosed, treated late or treated with ineffective drugs can be fatal (16). Approximately 2–4% of acute typhoid cases result in the development of a chronic carrier state (17). These non-symptomatic carriers are the natural reservoir for *S. Typhi* and can serve to maintain the disease in its endemic state or to directly infect individuals (15).

Virulent strains of *S. Typhi* upon ingestion are able to pass through the stomach acid barrier, colonize the intestinal tract, penetrate the lumen and enter the lymphatic system and blood stream, thereby causing disease. One possible mechanism by which disease may be prevented is by evoking a local immune response in the intestinal tract. Such local immunity may be induced by oral ingestion of a live attenuated strain of *S. Typhi* undergoing an aborted infection. The ability of *S. Typhi* to cause disease is dependent upon the bacteria possessing a complete lipopolysaccharide (13). The *S. Typhi* Ty21a vaccine strain, by virtue of a reduction in enzymes essential for lipopolysaccharide biosynthesis, is restricted in its ability to produce complete lipopolysaccharide (13,14).

Indications and Usage

Vivotif® (Typhoid Vaccine Live Oral Ty21a) is indicated for immunization of adults and children greater than 6 years of age against disease caused by *Salmonella Typhi*. Selective immunization against typhoid fever is recommended for the following groups: 1) travellers to areas in which there is a recognized risk of exposure to *S. Typhi*, 2) persons with intimate exposure (e.g. household contact) to a *S. Typhi* carrier, and 3) microbiology laboratorians who work frequently with *S. Typhi* (16).

Not all recipients of Vivotif® will be fully protected against typhoid fever. Vaccinated individuals should continue to take personal precautions against exposure to typhoid organisms. The vaccine will not afford protection against species of Salmonella other than Salmonella Typhi or other bacteria that cause enteric disease. The vaccine is not suitable for treatment of acute infections with S. typhi.

Information for Participants

It is essential that all 3 doses of vaccine are taken at the prescribed alternate day interval to obtain a maximal protective immune response. Vaccine potency is dependent upon storage under refrigeration [between 2 °C and 8 °C (35.6 °F– 46.4 °F)]. The vaccine should be stored under refrigeration at all times. It is essential to replace unused vaccine in the refrigerator between doses. The vaccine capsule should be swallowed approximately 1 hour before a meal with a cold or lukewarm [temperature not to exceed body temperature, e.g., 37 °C (98.6 °F)] drink. Care should be taken not to chew the vaccine capsule. The vaccine capsule should be swallowed as soon after placing in the mouth as possible.

Not all recipients of Vivotif® (Typhoid Vaccine Live Oral Ty21a) will be fully protected against typhoid fever. Travellers should take all necessary precautions to avoid contact or ingestion of potentially contaminated food or water. Several anti-malaria drugs, such as mefloquine, chloroquine and proguanil possess antibacterial activity which may interfere with the immunogenicity of Vivotif®. Clinical results indicate that mefloquine and chloroquine can be administered together with Vivotif®. Proguanil should be administered only if 10 days or more have elapsed since the final dose of Vivotif® was ingested. Any SAE related to the administration of the vaccine should be reported to a health care provider.

Carcinogenesis, Mutagenesis, Impairment of Fertility
Long-term studies in animals with Vivotif® have not been performed to evaluate carcinogenic potential, mutagenic potential or impairment of fertility.

Pregnancy
Category C
Animal reproduction studies have not been conducted with Vivotif®. It is not known whether Vivotif® can cause fetal harm when administered to pregnant women or can affect reproduction capacity. Vivotif® should be given to a pregnant woman only if clearly needed.

Nursing Mothers
There is no data to warrant the use of this product in nursing mothers. It is not known if Vivotif® is excreted in human milk.

Pediatric Use
The safety and efficacy of Vivotif® has not been established in children less than 6 years of age. This product is not indicated for use in children less than 6 years of age.

Dosage and Administration

One capsule is to be swallowed approximately 1 hour before a meal with a cold or lukewarm [temperature not to exceed body temperature, e.g., 37 °C (98.6 °F)] drink on alternate days, e.g., days 1, 3 and 5. Immunization (ingestion of all 3 doses of Vivotif®, Typhoid Vaccine Live Oral Ty21a) should be completed at least 1 week prior to potential exposure to *S. typhi*.

The blister containing the vaccine capsules should be inspected to ensure that the foil seal and capsules are intact. The vaccine capsule should not be chewed and should be swallowed as soon after placing in the mouth as possible. A complete immunization schedule is the ingestion of 3 vaccine capsules as described above.

Re-immunization
The optimum booster schedule for Vivotif® has not been determined. Efficacy has been shown to persist for at least 5 years. Further, there is no experience with Vivotif® as a booster in persons previously immunized with parenteral typhoid vaccine. It is recommended that a re-immunization dose consisting of 3 vaccine capsules taken on alternate days be given every 5 years under conditions of repeated or continued exposure to typhoid fever (16).

How Supplied

A single foil blister contains 3 doses of vaccine in a single package.

Storage

Vivotif® (Typhoid Vaccine Live Oral Ty21a) is not stable when exposed to ambient temperatures. Vivotif® should therefore be shipped and stored between 2 °C and 8 °C (35.6 °F–46.4 °F). Each package of vaccine shows an expiration date. This expiration date is valid only if the product has been maintained at 2 °C–8 °C (35.6 °F–46.4 °F).

Manufactured by

PaxVax Berna
Oberriedstrasse 68
CH-3174 Thörishaus
Switzerland

Distribuited by

Alloga AG
Buchmattstrasse 10
3400 Burgdorf

3.3 Preclinical Evidence

The present study evaluates specific immune responses induced by the commercial vaccine Vivotif®. Safety and efficacy of this vaccine have been demonstrated in several clinical studies and post-marketing studies (please refer to section 3.4). Therefore no preclinical evidence is presented in this protocol.

3.4 Clinical Evidence to Date

Results from clinical studies indicate that adults and children greater than 6 years of age may be protected against typhoid fever following the oral ingestion of 3 doses of Vivotif® (Typhoid Vaccine Live Oral Ty21a). The efficacy of the *S. typhi* Ty21a strain has been evaluated in a series of randomized, double-blind, controlled field trials. Suspected typhoid cases, detected by passive surveillance, were confirmed bacteriologically either by blood or bone marrow culture.

The first trial was performed in Alexandria, Egypt with a study population of 32,388 children aged 6 to 7 years. 3 doses of vaccine, in the form of a freshly reconstituted suspension administered after ingestion of 1 g of bicarbonate, were given on alternate days. Immunization resulted in a 95% decrease [95% confidence interval (CI) = 77%–99%] in the incidence of typhoid fever over a 3-year period of surveillance (18).

A series of field trials were subsequently performed in Santiago, Chile, to evaluate efficacy when the vaccine strain was administered in the form of an acid-resistant enteric-coated capsule. The initial trial involved 82,543 school-aged children, and compared 1 or 2 doses of vaccine given one week apart. After 24 months of surveillance vaccine efficacy was 29% (95% CI = 4%–47%) for the single dose schedule and 59% (95% CI = 41%–71%) for the 2-dose schedule (19). A further field trial was performed in Santiago, Chile, involving 109,594 school-aged children (20). Three doses of enteric-coated capsules were administered either on alternate days (short immunization schedule) or 21 days apart (long immunization schedule). Following 36 months of surveillance vaccination resulted in a 67% (95% CI = 47%–79%) decrease in the incidence of typhoid fever in the short immunization schedule group and a 49% reduction (95% CI = 24%–66%) in the long immunization schedule group. After 48 months of surveillance the short immunization schedule resulted in a 69% (95% CI = 55%–80%) decrease in typhoid fever (21). An undiminished level of protection was observed during the fifth year of surveillance. A field trial was next conducted in Santiago, Chile, to determine the relative efficacy of 2, 3 and 4 doses of enteric-coated vaccine administered on alternate days to school-aged children. Relative vaccine efficacy as determined by comparison of disease incidence within the 3 vaccinated groups was highest for the 4 dose regimen (22). The incidence of typhoid fever per 105 study subjects

was 160.5 (95% CI = 130–191) for the 3 dose regimen versus 95.8 (95% CI = 71–121) for the 4 dose regimen ($p<0.004$). An additional field trial to determine vaccine efficacy was conducted in Plaju, Indonesia, involving 20,543 individuals approximately 3 to 44 years of age (23). Due to logistical considerations 3 doses of enteric-coated capsules were administered at weekly intervals, a schedule known to provide suboptimal protection (20). After 30 months of surveillance vaccine efficacy for all age groups was 42% (95% CI = 23%–57%). Vaccine organisms can be shed transiently in the stool of vaccine recipients (24). However, secondary transmission of vaccine organisms has not been documented. Ty21a has not been isolated from blood cultures following immunization.

At present, the precise mechanism(s) by which Vivotif® confers protection against typhoid fever is unknown. However, it is known that immunization of adult subjects can elicit a humoral anti-*S. Typhi* LPS antibody responses. Taking advantage of this fact, the seroconversion rate (defined as a ≥0.15 increase in optical density units over baseline determined in an ELISA) was compared in an open study between adults living in an endemic area (Chile) and non-endemic areas (United States and Switzerland) after the ingestion of 3 doses of vaccine. Comparable seroconversion rates were seen between these groups (25).

Adverse Reactions

More than 1.4 million doses of Ty21a have been administered in controlled clinical trials and more than 150 million doses of Vivotif® (Typhoid Vaccine Live Oral Ty21a) have been marketed world-wide. Active surveillance for adverse reactions of enteric-coated capsules was performed in a pilot study (22) and in a subgroup of a large field trial involving a total of 483 individuals receiving 3 vaccine doses. The overall symptom rates from both studies when vaccinated with capsules were combined and shown to be: abdominal pain (6.4%), nausea (5.8%), headache (4.8%), fever (3.3%), diarrhea (2.9%), vomiting (1.5%) and skin rash (1.0%). Only the incidence of nausea occurred at a statistically higher frequency in the vaccinated group as compared to the placebo group (23). Administration of vaccine doses more than 5-fold higher than the currently recommended dose caused only mild reactions in an open study involving 155 healthy adult males (24).

Post-marketing surveillance has revealed that adverse reactions are infrequent and mild. Adverse reactions reported to the manufacturer during 1991–1995, during which time over 60 million doses (capsules) were administered included: diarrhea (N = 45), abdominal pain (N = 42), nausea (N = 35), fever (N = 34), headache (N = 26), skin rash (N = 26), vomiting (N = 18), or urticaria on the trunk and/or extremities (N = 13). One isolated, non-fatal anaphylactic shock considered to be an allergic reaction to the vaccine was reported.

3.5 Dose Rationale

Parameters as dose of vaccine, route of administration, regimen employed and dosage period used in this study, correspond to those used for the administration of the commercial vaccine and are summarized in section 3.2.

3.6 Explanation for choice of comparator (or placebo)

Healthy untreated volunteers serve as a control group.

3.7 Risks / Benefits

Vivotif® is indicated for immunization of adults and children greater than 6 years of age. This vaccine protects against disease caused by *Salmonella Typhi*, as demonstrated in several clinical studies (refer to section 3.4). Selective immunization against typhoid fever is recommended for the following groups: 1) travellers to areas in which there is a recognized risk of exposure to *S. Typhi*, 2) persons with intimate exposure (e.g. household contact) to a *S. Typhi* carrier, and 3) microbiology laboratorians who work frequently with *S. Typhi*. This vaccine has been shown to be safe; adverse reactions during

administration of enteric-coated capsules of Vivotif® are described in section 3.4. Notably, during a large field trial only the incidence of nausea occurred at a statistically higher frequency in a vaccinated group in comparison with the placebo group.

Despite demonstrated safety of Vivotif®; the vaccine will not be administered to persons during an acute febrile illness. Safety of the vaccine has not been demonstrated in persons deficient in their ability to mount a humoral or cell-mediated immune response, due to either a congenital or acquired immunodeficient state including treatment with immunosuppressive or antimitotic drugs. Therefore the vaccine will not be administered to these persons.

3.8 Justification of choice of study population

The study population was selected considering the prescription indications for Vivotif® (please refer to section 3.2). To minimize the risk of adverse events (AE), a population with an age ≥18 and ≤50 years was selected. Additional inclusion criteria important to ensure the welfare of the study subjects were selected and include: No use of an immune modulator in the past year, no use of systemic corticosteroid treatment in the past 30 days and having regular bowel movement. Exclusion criteria important to preserve the health of the subjects included in the study are: positive HIV serology or any immune deficiency, current or planned pregnancy, breastfeeding and obstipation.

4. STUDY OBJECTIVES

4.1 Overall Objective

The purpose of this study is to evaluate the specific immune responses against *Salmonella* porins elicited by the commercial vaccine Vivotif®, as well as the genetic modifications of the live attenuated bacteria contained in the vaccine formulation occurring during vaccination.

4.2 Primary Objective

The primary objective of this study is to evaluate the porins-specific T and B cell immune responses after vaccination with the commercial live attenuated *Salmonella* vaccine Vivotif®.

4.3 Secondary Objectives

The secondary objective is to evaluate the presence of bacteria that bear mutations in their DNA sequences in comparison with bacteria from the original inoculum administered during vaccination.

4.4 Safety Objectives

Not applicable

5. STUDY OUTCOMES

5.1 Primary Outcome

The primary endpoints of this study are: antibody levels of IgM and IgG specific against porins in serum and IgA in stool, number of porins-specific T cells from blood and amount of porins-specific B cells in blood. These parameters reflect the specific cellular and humoral immune responses induced during vaccination and will allow the study of these types of responses in the present study. Time points for evaluation of primary outcomes are described in section 9.3.

5.2 Secondary Outcomes

The secondary outcomes of this study are the bacteria that bear mutations in their DNA sequences in comparison with bacteria from the original inoculum administered during vaccination. This parameter will be assessed throughout the intervention and follow-up period as depicted in table of the study schedule.

5.3 Other Outcomes of Interest

Not applicable.

5.4 Safety Outcomes

Not applicable.

6. STUDY DESIGN

6.1 General study design and justification of design

This is an open, monocentric, controlled, randomized clinical study conducted with healthy volunteers to evaluate anti-*Salmonella* porins specific immune responses after vaccination with the commercial live attenuated *Salmonella* vaccine Vivotif®. 15 subjects will be vaccinated with Vivotif® and 5 subjects will be left untreated. The study will consist of 7 visits that in total will last 67 days. For a general description of the times in which every assessment will be performed please refer to table 1 in section 9.1. For a description of the procedures performed in the different phases of the study please refer to section 9.3.

The results obtained from this study will reflect the specific cellular and humoral immune responses against *Salmonella Typhi* porins induced during vaccination. The study was designed to have the minimum number of visits possible to increase the probability of study completion by the participants. Enough time of follow up after vaccination is given to analyse the specific anti-porins immune responses in a timeframe that allows collecting solid data.

From the screening of the first participant to the end of follow up, the study will last approximately 3 months. The first participant-in is expected to be in June, 2015 and the last participant-out is expected for September, 2015. End of trial is expected by November, 2015.

6.2 Methods of minimising bias

6.2.1 Randomisation

Randomization sequence will be created with a 1:3 allocation using random block sizes of 4.

6.2.2 Blinding procedures

Not applicable.

6.2.3 Other methods of minimising bias

In order to minimize bias block randomization will be used.

6.3 Unblinding Procedures (Code break)

Not applicable.

7. STUDY POPULATION

Healthy volunteers permanent residents in the Canton of St Gallen and employees at the Kantonsspital St. Gallen (excluding the personnel from the Division of Infectious Diseases and Hospital Epidemiology or Institute of Immunobiology) will be recruited. All the data regarding the study subjects will be collected in the Division of Infectious Diseases and Hospital Epidemiology. Analysis of participants' samples will be done in the Institute of Immunobiology KSSG and Institute of Microbiology from ETH Zürich.

7.1 Eligibility criteria

Participants fulfilling all of the following <u>inclusion</u> criteria are eligible for the study:

- Ability to understand the experimental nature of the vaccine evaluation and the patient informed consent form
- Written informed consent documented by date and signature to be obtained prior to any study specific procedure
- Age 18-50 years old
- Regular bowel movement (1+ defecation per day)
- Willingness to adhere to the strict timing schedule for the study evaluation
- Willingness to provide stool and blood samples in the indicated visits

The presence of any one of the following <u>exclusion</u> criteria will lead to exclusion of the participant:

- Previous use of an oral vaccine against *Salmonella* in the past 3 years
- Gastrointestinal infection caused by any *Salmonella* species during the past 3 years
- Positive HIV serology or any immune deficiency
- Current or planned pregnancy during the course of the study
- Unwillingness to use at least one method of birth control in women of childbearing age during the course of the study
- Breastfeeding
- Obstipation
- Hypersensitivity to any component of the vaccine or the enteric-coated capsule
- Use of an immune modulator in the past year
- Use of systemic corticosteroid treatment in the past 30 days
- Use of antibiotics within 1 week preceding and during the present study
- Current use of proton-pump inhibitors
- Participation in another study with investigational drug within the 30 days preceding and during the present study

7.2 Recruitment and screening

Participants will be recruited in the Division of Infectious Diseases and Hospital Epidemiology, Kantonsspital St. Gallen by PD Dr. Werner Albrich. Employees at the Kantonalsspital St Gallen (excluding the personnel from the Division of Infectious Diseases and Hospital Epidemiology and Institute of Immunobiology) and permanent residents in the Canton of St. Gallen will be recruited. All the participants are independent of the persons involved in the trial. A flyer for the recruitment of participants will be submitted and approved by the EC, and will be used during the trial. New employers including medical students (Unterassistenten) will be informed during their entry examination by the Employee Health (Personalärztlicher Dienst) or during introductory sessions by

Human Resources.

At first, informed consent will be obtained. Participants will be allowed an appropriate period of reflection to ensure that informed consent is obtained through a communicative process. After participant consent, information regarding medical history and information used to apply inclusion and exclusion criteria will be collected. A physical examination and measurement of the vital signs to evaluate the overall health status of the study subjects will be made. Pregnancy and HIV tests will be performed at the beginning of the study. Blood samples will be collected and stored for evaluation of immunological parameters before the first vaccine dose administration.

Participants who fulfil inclusion criteria and are enrolled in the study will receive an inconvenience compensation of 50 CHF per study visit at the end of the study, which includes transportation costs.

7.3 Assignment to study groups

Block randomization will be used in this study. Random number tables will be generated and assignments will be enclosed in sequentially numbered, opaque, sealed envelopes to designate the 15 subjects that will receive the vaccine and the 5 subjects that will not be administered. After assignment to vaccination/not vaccination groups, the participants will be informed in the second study visit to which group they belong by personnel from the Division of Infectious Diseases and Hospital Epidemiology, Kantonsspital St. Gallen.

7.4 Criteria for withdrawal / discontinuation of participants

Study participants are withdrawn from the study in the following cases: a) withdrawal of informed consent, b) non-compliance and c) presence of SAE due to an unknown health condition of the participants or any other cause that compromise the integrity of participants.

8. STUDY INTERVENTION

8.1 Identity of Investigational Products (treatment)

8.1.1 Experimental Intervention (treatment)

Vivotif® (Typhoid Vaccine Live Oral Ty21a) is a live attenuated vaccine for oral administration only. The vaccine contains the attenuated strain *Salmonella Typhi* Ty21a (1,2). Vivotif® is a commercial and Swissmedic-approved drug. It is manufactured by PaxVax Berna. The vaccine strain is grown in fermenters under controlled conditions in medium containing a digest of yeast extract, an acid digest of casein, dextrose and galactose. The bacteria are collected by centrifugation, mixed with a stabilizer containing sucrose, ascorbic acid and amino acids, and lyophilized. The lyophilized bacteria are mixed with lactose and magnesium stearate and filled into gelatin capsules which are coated with an organic solution to render them resistant to dissolution in stomach acid. The contents of each enteric-coated capsule are shown in Table 1.

Table 1: Contents of one enteric-coated capsule of Vivotif® (Typhoid Vaccine Live Oral Ty21a)
Viable *S. Typhi* Ty21a 2.0–10.0x10₉ colony-forming units*
Non-viable *S. Typhi* Ty21a 5–50x10₉ bacterial cells
Sucrose 3.3 – 34.2 mg
Ascorbic acid 0.2 – 2.4 mg
Amino acid mixture 0.3 – 3.0 mg
Lactose up to 180 - 200 mg
Magnesium stearate 3.6–4.0 mg
*Vaccine potency (viable cell counts per capsule) is determined by inoculation of agar plates with appropriate dilutions of the vaccine suspended in physiological saline.

8.1.2 Control Intervention (standard/routine/comparator treatment)

Not applicable.

8.1.3 Packaging, Labelling and Supply (re-supply)

The enteric-coated, salmon/white capsules of Vivotif® are packaged in 3-capsule foil blisters in a single package for distribution. Each package of vaccine shows an expiration date. This expiration date is valid only if the product has been maintained at 2 °C–8 °C (35.6 °F–46.4 °F).

The product used in the study will be obtained directly from the distributor Alloga AG, Buchmattstrasse 10, 3400 Burgdorf and will be kept at 4°C in a secured place in the study location. The study product will be labelled in accordance to Annex 13, Volume 4 of EUDRALEX and local regulatory requirements. The following label information will be added to each package of the product:

a) Sponsor
b) Patient identification number
c) Study number
d) Co-Investigator and study site

Unused vaccine: (Partly) unused or expired medication will be destroyed in the study site according to local guidelines but only after approval by the sponsor-investigator. The destruction shall be documented.

8.1.4 Storage Conditions

Vivotif® (Typhoid Vaccine Live Oral Ty21a) is not stable when exposed to ambient temperatures. Therefore it is shipped and stored between 2 °C and 8 °C (35.6 °F–46.4 °F).

8.2 Administration of experimental and control interventions

8.2.1 Experimental Intervention

3 doses of the vaccine are to be administered in alternate days (0, 2 and 4). Vaccine potency is dependent upon storage under refrigeration [between 2 °C and 8 °C (35.6 °F– 46.4 °F)]. The vaccine should be stored under refrigeration at all times. One capsule is to be swallowed approximately 1 hour before a meal with a cold or lukewarm [temperature not to exceed body temperature, e.g., 37 °C (98.6 °F)] drink. Care should be taken not to chew the vaccine capsule. The vaccine capsule should be swallowed as soon after placing in the mouth as possible.

8.2.2 Control Intervention

Not applicable.

8.3 Dose / Device modifications

Post-marketing surveillance has revealed that adverse reactions are infrequent and mild. Adverse reactions reported to the manufacturer during 1991–1995, time in which over 60 million doses (capsules) were administered included: diarrhea (N = 45), abdominal pain (N = 42), nausea (N = 35), fever (N = 34), headache (N = 26), skin rash (N = 26), vomiting (N = 18), or urticaria in the trunk and/or extremities (N = 13). One isolated, non-fatal anaphylactic shock considered to be an allergic reaction to the vaccine was reported. This adverse reactions show that the vaccine is relatively safe and therefore the expected adverse reactions will be infrequent and mild as described above.

Nevertheless in case of any SAE or in direct request from a participant to withdraw from the study, the vaccine administration will be stopped and the appropriate medication/treatment will be applied until the complete recovery of the study subjects.

8.4 Compliance with study intervention

Non-compliance in the study is defined as any deviation in the participants' behaviour from that recommended by the doctor, included but not limited to: a) Attendance to study visits, b) Willingness to provide blood samples, c) Delivery of stool samples in the indicated visits, d) Avoiding ingestion of drugs, foods and any substance specified by the doctor during the study period and e) Attendance for further investigation or follow up.

Adherence to the intervention will be ensured by administration of the vaccine during study visits in the Division of Infectious Diseases and Hospital Epidemiology, Kantonsspital St. Gallen. Patients will receive a phone call one day previous to each study visit as a reminder. All blood samples will be taken at the study site in the corresponding visits. Sterile containers will be provided to study subjects to collect stool samples before attending to the next visit when the samples will be received. In case that the participant forgets to bring the stool sample in the corresponding visit, the sample can be taken during the study visit. A list of drugs, foods and substances not allowed to ingest during the study will be delivered to study subjects, and a detailed explanation of the importance to adhere to the

doctor's indications will be given. Please refer to section 8.8 for details about drug accountability.

8.5 Data Collection and Follow-up for withdrawn participants

If a subject withdraws during the interventional phase of the study and does not consent to continued follow-up of associated clinical outcome information, the investigator will not access, for purposes related to the study, the subject's medical record or other confidential records requiring the subject's consent.

If a subject withdraws during the interventional phase of the study but agrees to continue with the follow up of associated clinical outcome information, the following measures for data collection will be taken depending on the time point of withdrawal: a) Data from anti-porins specific immune responses will only be included in the analysis when the participant withdraws after visit 5, and b) Data from study subjects withdrawn at early time points will only be used for the genetic analysis of live attenuated bacteria contained in the formulation (applicable for study subjects that withdraw after visit 3).

8.6 Trial specific preventive measures

The following preventive measures will be implemented:

- Assessment of participants' health and suitability to participate in the study before starting of the intervention by means of: a) pregnancy test, c) HIV test and c) Physical examination.
- Avoid the use of the following medications during the clinical trial: corticosteroids, immune modulators, antimitotic drugs, proton-pump inhibitors, sulphonamides, antibiotics and anti-malaria drugs, such as mefloquine, chloroquine and proguanil.
- Avoid taking the vaccine during an acute febrile illness or gastrointestinal illness and if persistent diarrhea or vomiting is occurring. Safety of the vaccine has not been demonstrated in persons deficient in their ability to mount a humoral or cell-mediated immune response, due to either a congenital or acquired immunodeficient state including treatment with immunosuppressive or antimitotic drugs.

Rescue medication: *Salmonella* gastrointestinal infections usually resolve in 5-7 days and most do not require treatment other than oral fluids. Persons with severe diarrhea may require rehydration with intravenous fluids. Choices for antibiotic therapy for severe infections include fluoroquinolones, third-generation cephalosporins, and ampicillin.

8.7 Concomitant Interventions (treatments)

The concomitant administration of yellow fever vaccine does not suppress the immune response elicited by the Ty21a vaccine strain. There is no data regarding simultaneous administration of other parenteral vaccines or immunoglobulins with Vivotif®. Nevertheless during the study it is not recommended to take any additional treatment unless necessary and with prescription from a doctor.

8.8 Study Drug Accountability

The medication provided for this study is for clinical trial use only as outlined in the protocol. It is the Investigator's responsibility to establish a system for handling study treatments, including investigational medicinal products, at the study site to ensure that:

- Deliveries of study medication are correctly received by a responsible person
- Such deliveries are recorded, signed by an authorised person and documentation kept
- Study medication is handled and stored safely and properly as stated on the label and the protocol
- Study medication are only dispensed to study subjects in accordance with the protocol
- Drug accountability logs (as provided by the Sponsor) shall be maintained by the study sites to record drug inventory (recording batch number, expiry date, quantities and dates medication received, dispensed and /or destroyed by the study site) continuously
- Study medication records and logs shall be maintained properly throughout the study.

8.9 Return or Destruction of Study Drug

At the end of the trial, the study drug will be destroyed in accordance with standard procedures or returned to the sponsor.

9. STUDY ASSESSMENTS

9.1 Study flow chart(s) / table of study procedures and assessments

Table 1. The following table summarizes the flow of the study, specifying the time frame between each visit and a general description of the procedures to be performed. For a detailed description of each study visit please refer to section 6.1. For a specific description of the assessments to be performed please refer to table 2 in section 9.2.3.

Study Periods	Screening	Vaccine administration (intervention period)					
Visit[1]	1	2	3	4	5	6	7
Time (day)	-7 to -1	0	2	4	11 ± 1	25 ± 1	60 ± 1
Patient Information and Informed Consent	X						
Medical History	X						
In- /Exclusion Criteria	X						
Physical Examination	X						
Vital Signs	X	X	X	X	X	X	X
Pregnancy Test	X						
Vivotif® vaccination[2]		1st dose	2th dose	3rd dose			
Blood sample collection		X			X	X	X
Stool sample collection[3]		X	X	X	X	X	X
Serious adverse events		X	X	X	X	X	X

[1]Patients will receive a phone call one day prior to each study visit as a reminder. [2]Vaccine will be administered in the study site during the indicated study visits. [3]Stool samples will be received at the study site. Sterile containers are provided to the participants in the preceding study visit (i.e. sample collected at study visit number 2, will be obtained by the participant in a sterile container provided in study visit number 1). *It is very important to indicate to the patient that the stool sample should be obtained during the same day of the study visit and must be stored at 4°C until delivered to the personnel from the study*. In case that the participant forgets to bring the stool sample in the corresponding visit, the sample can be taken during the study visit.

9.2 Assessments of outcomes

9.2.1 Assessment of primary outcome

The primary endpoints of this study are: a) antibody levels of IgM and IgG specific against porins in serum (during visits 2, 5, 6 and 7) and IgA in stool (during visits 2, 5, 6 and 7), b) number of porins-specific T cells from blood (during visits 2, 5, 6 and 7) and c) amount of porins-specific B cells in blood (during visits 2, 5, 6 and 7) and are described below.

- IgM and IgG levels in serum and IgA in stool samples will be determined by the enzyme-linked immunosorbent assay (ELISA), which is a common laboratory technique used to measure the concentration of antibodies or antigens within a sample. The basic ELISA is distinguished from other antibody-based assays because separation of specific and non-specific interactions occurs via serial binding to a solid surface, usually a polystyrene plate, and because quantitative results can be achieved. The steps of the ELISA result in a colored end product which intensity correlates to the amount of antigen present in the original sample. ELISAs are quick and simple to carry out, and since they are designed to rapidly handle a large numbers of samples in parallel, they are a very popular choice for the evaluation of various research and diagnostic targets.

The type of ELISA used in this study is a direct assay in which a standard amount of the antigen (*Salmonella Typhi* purified porins) will be fixed to polystyrene plates, followed by incubation with serum samples (for blood antibodies detection) or a preparation of stool samples. Incubation with antibodies against IgM and IgG (for blood samples) and IgA (for stool samples) coupled to an enzyme is followed and a substrate for this enzyme is then added. A colorimetric substrate-enzyme reaction occurs. This color change shows that the secondary antibody has bound to the primary antibody, which strongly implies that the donor had a specific immune reaction against the antigen (*Salmonella Typhi* porins). A spectrometer is used to give quantitative values for color strength and measurement of specific antibody levels can be determined.

- Porins-specific T cells from blood will be quantified by flow cytometry. The use of this technology allows for the analysis of highly defined subsets of cells with unique phenotypes and functions. In this assay peripheral blood mononuclear cells (PBMC) are isolated by standard density centrifugation. Cells are *in vitro* cultured and stimulated with specific peptides from *Salmonella Typhi* porins, followed by staining of specific markers to analyse T cell populations by flow cytometry. The numbers of T helper cells (CD4+) or cytotoxic T cells (CD8+) that produce IFN-γ upon antigen stimulation (activated T cells) are detected.

- Quantification of porins-specific B cells in blood by ELISPot. The enzyme-linked immunospot (ELISpot) assay is a highly sensitive immunoassay that measures the frequency of antibody-secreting cells at the single-cell level. Cells from blood are cultured on a surface coated with *Salmonella* porins. Antibodies that are secreted by the cells bind to the surface and after an appropriate incubation time cells are removed and detection is made using a biotinylated antibody. A streptavidin-enzyme conjugate is used as a secondary antibody and a precipitating substrate is added to allow the detection of visible spots on the surface. Each spot corresponds to an individual antibody-secreting cell.

These parameters reflect the specific cellular and humoral immune responses induced during vaccination and will allow the study of these types of responses in the present study. Blood and stool samples used for these analyses will be obtained during the study visits indicated in table 1. For a detailed description of each study visit please refer to section 9.3. For a specific description of the assessments to be performed please refer to table 2 in section 9.2.2.

9.2.2 Assessment of secondary outcomes

The secondary outcomes of this study are the mutants from live attenuated *Salmonella* in stool after vaccination (during visits 2, 3, 4 and 5). For this, bacterial culture of stool samples will be performed in specific culture mediums, followed by DNA extraction and amplification by Polymerase Chain Reaction (PCR). The genome of *Salmonella* will be then sequenced to analyse mutations in the original sequences of the bacterial populations.

9.2.3 Assessment of other outcomes of interest

Table 2. Assessments performed during the study visits.

Procedure/Assessment	Description
Patient information and Informed Consent (IC) - Visit number 1 after obtaining the ICF	Informed consent will be obtained in accordance with GCPs. Appropriate written information (content and wording) that supports the participants to understand the clinical trial will be used. Prior to participation in the trial, the subject will receive a copy of the signed and dated written informed consent form. During participation in the trial, the subject will receive a copy of the signed and dated consent form updates and a copy of any amendments to the written information provided.
Medical history – Visit number 1 after obtaining the ICF	The following information will be collected from every participant in a discussed questionnaire during the screening visit:<br>- Any current ongoing illness<br>- Any previous illness<br>- Any previous surgery/operation<br>- Family diseases<br>- Childhood diseases<br>- Social history including: occupation, recent foreign travel and exposure to environmental pathogens through recreational activities or food ingestion<br>- Regular and acute medications<br>- Allergies to medications, foods, environmental factors and specific materials
In- /exclusion criteria – Visit number 1 after obtaining the ICF	Information concerning inclusion and exclusion criteria will be collected during the screening visit. To review inclusion and exclusion criteria please refer to section 7.1.
Physical examination – Visit number 1 after obtaining the ICF	Participant information that will be obtained includes: name, age, height and weight. Main organ systems will be investigated by inspection, palpation, percussion, and auscultation in the indicated study visits in table 1 from section 9.1. In all other visits a limited and symptom oriented physical examination is performed if clinically indicated. Any abnormalities will be registered in the Case Report Form (CRF).
Vital signs – Visit number 1 after obtaining the ICF	Body temperature, heart rate and blood pressure will be measured and will be recorded as indicated on the visit assessment schedule. Any abnormalities will be registered in the CRF.
Pregnancy test – Visit number 1 after obtaining the ICF	A standard urine pregnancy test that detects the presence of human chorionic gonadotropin (hCG) will be used. Sterile containers will be provided to study subjects to collect the urine sample during the first visit. The test will be performed at the facilities of the Division of Infectious Diseases and Hospital Epidemiology, Kantonsspital St. Gallen.
HIV test - Visit number 1 after obtaining the ICF	During visit number 1 (screening visit), a drop of blood will be obtain using a lancet and a standard rapid HIV test that detects antibodies against the virus will be used.
Vivotif® vaccination – Visits 2, 3 and 4	Parameters as dose of vaccine, route of administration, regimen employed and dosage period used in this study, correspond to those used for the administration of the commercial vaccine and are summarized in section 3.2.

	Vivotif® vaccine will be applied at least one hour before meal in the study visits number 2, 3 and 4 as indicated in table number 1 from section 9.1. The vaccine will be administrated at the facilities of the Division of Infectious Diseases and Hospital Epidemiology, Kantonsspital St. Gallen.
Blood sample collection – Visits 2, 5, 6 and 7	Blood sample collection will take place at the Division of Infectious Diseases and Hospital Epidemiology, Kantonsspital St. Gallen. Samples will be sent to the Institute of Immunobiology, Kantonsspital St. Gallen where will be frozen and used to evaluate specific anti-porins immune responses. The last meal should be at least 2 hours before the blood sample collection. Specimens will be transported within 1 hour after phlebotomy. Samples will be stored at -80°C unless they are immediately processed.
Stool sample collection – Visits 2, 3, 4, 5, 6 and 7	Sterile containers will be provided to study subjects to collect stool samples before attending to the next visit when the samples will be obtained at the Division of Infectious Diseases and Hospital Epidemiology, Kantonsspital St. Gallen. In case that the participant forgets to bring the stool sample in the corresponding visit, the sample can be taken during the study visit.<br>One gram of the stool sample from each participant will be deposited in a screw cap tube containing 1ml of sterile PBS solution (sample A) and one gram of the stool sample from each participant will be deposited in a screw cap tube containing 1ml of sterile PBS solution, 10% glycerol (sample B). Samples will be transferred at 4°C to the Institute of Immunobiology, Kantonsspital St. Gallen and will be frozen at -80°C. Sample A will be used to assess the presence of specific IgA antibodies against porins (only samples obtained during study visits 2, 5, 6 and 7) and sample B will be transferred (at -80°C) to the Institute of Microbiology, ETH Zurich to evaluate the genetic changes of live attenuated bacteria contained in the formulation (only samples obtained during study visits 2, 3, 4 and 5).
Serious adverse events (SAE) - Visits 2, 3, 4, 5, 6 and 7	Only SAE will be reported to Ethics Committee according to Swiss regulations. Reports will start from the moment of the first vaccine dose administration and will last until the end of the study.

For a general description of the times in which every assessment will be performed please refer to table 1 in section 9.1. For a detailed description of each study visit please refer to section 6.1.

9.2.4 Assessment of safety outcomes

9.2.4.1 Serious adverse events

Recording of information about adverse reactions during the study includes:

- Time of onset
- Duration
- Resolution
- Action to be taken
- Assessment of intensity
- Relationship with study treatment

For definition and procedures please refer to section 10. All SAE detected by the medical personnel of the Division of Infectious Diseases and Hospital Epidemiology, Kantonsspital St. Gallen will be attended immediately and reported on a timely basis to the EC. During study visits participants will be examined and questioned by the medical personnel about their health status. In case of any SAE a questionnaire will be made by the medical personnel including all points described above. Spontaneous reports will also be made in case a SAE occurs and the participants attend for medical attention outside the study visits time frame.

9.2.4.2 Vital signs

Body temperature, heart beat and blood pressure will be monitored in every study visit.

9.2.5 Assessments in participants who prematurely stop the study

If a subject withdraws during the interventional phase of the study and does not consent to continued follow-up of associated clinical outcome information, the investigator will not access, for purposes related to the study, the subject's medical record or other confidential records requiring the subject's consent.

If a subject withdraws during the interventional phase of the study but agrees to continue with the follow up of associated clinical outcome information, the following measures for data collection will be taken depending on the time point of withdrawal: a) Data from anti-porins specific immune responses will only be included in the analysis when the patient withdraws after visit 5, and b) Data from patients withdrawn at early time points will only be used for the genetic analysis of live attenuated bacteria contained in the formulation (applicable for study subjects that withdrawn after visit 3).

9.3 Procedures at each visit

The screening visit should be performed within 7 days prior to visit number 2, when the first vaccine dose will be administrated. It is responsibility of the investigator to obtain written informed consent from each patient participating in this study after adequate face to face explanation of the aims, methods, objectives and potential hazards of the study. The patients who agree to participate in the study must sign the informed consent form during the screening visit prior to any study-related assessment or procedure.

9.3.1 Visit 1. Screening visit, day -7.

Recruitment of study subjects

Obtaining Informed Consent

Obtaining medical history and information used to apply inclusion and exclusion criteria

Physical examination and measurement of vital signs to evaluate the overall health status of the study subjects

Urine pregnancy test

HIV test

9.3.2 Visit 2. Intervention visit, day 0.

Enrolment in the study of participants that fulfil eligibility criteria (refer to section 7.1)

Measurement of vital signs

Blood sample collection to be used as a control before vaccination for the evaluation of specific anti-porins immune responses.

Stool sample collection to be used as control for the evaluation of anti-porins immune responses (determination of specific IgA antibodies agains *S. Typhi* porins) before the vaccine administration and as a control for the evaluation of genetic changes of live attenuated bacteria contained in the formulation.

Administration of first vaccine dose.

Reporting of SAE starts at this time point and lasts until the end of the study.

9.3.3 Visit 3. Intervention visit, day 2.

Measurement of vital signs

Second vaccine dose administration

Stool sample collection for evaluation of genetic changes of live attenuated bacteria contained in the formulation

9.3.4 Visit 4. Intervention visit, day 4.

Measurement of vital signs

Third vaccine dose administration

Stool sample collection for evaluation of genetic changes of live attenuated bacteria contained in the formulation

9.3.5 Visit 5. Intervention visit, day 6.

Measurement of vital signs

Blood sample collection for evaluation of specific anti-porins immune responses (day 7 after last immunization)

Stool sample collection for evaluation of genetic changes of live attenuated bacteria contained in the formulation and for determination of IgA antibodies (day 7 after last immunization)

9.3.6 Visit 6. Follow-up visit, day 27.

Measurement of vital signs

Blood sample collection for evaluation of specific anti-porins immune responses (day 21 after last immunization)

Stool sample collection for determination of IgA antibodies (day 21 after last immunization)

9.3.7 Visit 7. Follow-up visit, day 60.

Measurement of vital signs

Blood sample collection for evaluation of specific anti-porins immune responses (day 56 after last immunization)

Stool sample collection for determination of IgA antibodies (day 56 after last immunization)

10. SAFETY

10.1 Drug studies

The new law on clinical research (ordinance ClinO) does not require the documentation of adverse events (AEs) for Category A drug trials, therefore during the entire duration of the study only SAE will be collected, fully investigated and documented in source documents and case report forms (CRF). Study duration encompassed the time from when the participant signs the informed consent until the last protocol-specific procedure has been completed, including a safety follow-up period.

10.1.1 Definition and assessment of (serious) adverse events and other safety related events

An **Adverse Event** is any untoward medical occurrence in a patient or a clinical investigation participant administered a pharmaceutical product and which does not necessarily have a causal relationship with the study procedure. An AE can therefore be any unfavourable and unintended sign (including an abnormal laboratory finding), symptom, or disease temporally associated with the use of a medicinal (investigational) product, whether or not related to the medicinal (investigational) product. [ICH E6 1.2]

A **Serious Adverse Event** is classified as any untoward medical occurrence that:

- results in death,
- is life-threatening,
- requires in-patient hospitalization or prolongation of existing hospitalisation,
- results in persistent or significant disability/incapacity, or
- is a congenital anomaly/birth defect.

In addition, important medical events that may not be immediately life-threatening or result in death, or require hospitalisation, but may jeopardise the patient or may require intervention to prevent one of the other outcomes listed above should also usually be considered serious. [ICH E2A]

SAEs should be followed until resolution or stabilisation. Participants with ongoing SAEs at study termination (including safety visit) will be further followed up until recovery or until stabilisation of the disease after termination.

Assessment of Causality

Both Investigator and Sponsor-investigator make a causality assessment of the event to the study drug, based on the criteria listed in the ICH E2A guidelines:

Relationship	Description
Definitely	Temporal relationship<br>Improvement after dechallenge*<br>Recurrence after rechallenge<br>(or other proof of drug cause)
Probably	Temporal relationship<br>Improvement after dechallenge<br>No other cause evident
Possibly	Temporal relationship<br>Other cause possible
Unlikely	Any assessable reaction that does not fulfil the above conditions

Not related	Causal relationship can be ruled out
*Improvement after dechallenge only taken into consideration, if applicable to reaction	

Unexpected Adverse Drug Reaction

An "unexpected" adverse drug reaction is an adverse reaction, the nature or severity of which is not consistent with the applicable product information (e.g. Investigator's Brochure for drugs that are not yet approved and Product Information for approved drugs, respectively). [ICH E2A]

Suspected Unexpected Serious Adverse Reactions (SUSARs)

The Sponsor-Investigator evaluates any SAE that has been reported regarding seriousness, causality and expectedness. If the event is related to the investigational product and is both serious and unexpected, it is classified as a SUSAR.

Assessment of Severity

The severity grading scale used for this study will be in accordance to the grades for severity described in the "Common Terminology Criteria for Adverse Events v4.03 (CTCAE), June 14, 2010". Grade scales are summarized as follows:

Grade refers to the severity of the AE. The CTCAE displays Grades 1 through 5 with unique clinical descriptions of severity for each AE based on this general guideline:

Grade 1 Mild; asymptomatic or mild symptoms; clinical or diagnostic observations only; intervention not indicated.

Grade 2 Moderate; minimal, local or noninvasive intervention indicated; limiting age-appropriate instrumental ADL (instrumental Activities of Daily Living: refer to preparing meals, shopping for groceries or clothes, using the telephone, managing money, etc).

Grade 3 Severe or medically significant but not immediately life-threatening; hospitalization or prolongation of hospitalization indicated; disabling; limiting self-care ADL (self-care ADL: refer to bathing, dressing and undressing, feeding self, using the toilet, taking medications, and not bedridden).

Grade 4 Life-threatening consequences; urgent intervention indicated.

Grade 5 Death related to AE.

10.1.2 Reporting of serious adverse events and other safety related events

Reporting of SAEs

All SAEs must be reported immediately and within a maximum of 24 hours to the Sponsor-Investigator of the study. The Sponsor-Investigator will re-evaluate the SAE and return the form to the site.

SAEs resulting in death are reported to the local Ethics Committee (via local Investigator) within 7 days.

Reporting of SUSARs

A SUSAR needs to be reported to the local Ethics Committee (local event via local Investigator) within 7 days, if the event is fatal, or within 15 days (all other events).

Reporting of Safety Signals

All suspected new risks and relevant new aspects of known adverse reactions that require safety-related measures, i.e. so called safety signals, must be reported to the Sponsor-Investigator within 24 hours. The Sponsor-Investigator must report the safety signals within 7 days to the local Ethics Committee (local event via local Investigator).

Reporting and Handling of Pregnancies

Pregnant participants must immediately be withdrawn from the clinical study. Any pregnancy during the treatment phase of the study and within 30 days after discontinuation of study medication will be reported to the Sponsor-Investigator within 24 hours. The course and outcome of the pregnancy should be followed up carefully, and any abnormal outcome regarding the mother or the child should be documented and reported.

For all procedure regarding reporting of serious adverse events and other safety related events there will be Standard Operating Procedures (SOPs) in place.

10.1.3 Follow up of Serious Adverse Events

SAE will be reported from the moment starting the intervention until the end of the study to EC according to Swiss regulations. Participants terminating the study (either regularly or prematurely) with reported ongoing SAE, or vital signs being beyond the limit will be followed up until the end of the study (60 days after the administration of the first dose of the vaccine).

All SAE are collected, fully investigated and documented in the source document and appropriate CRF during the entire study period, i.e. from patient's informed consent until the last protocol-specific procedure, including a safety follow-up period. Documentation includes dates of event, treatment, resolution, assessment of seriousness and causal relationship to device and/or study procedure.

10.2 Medical Device Category C studies

Not applicable.

10.2.1 Definition and Assessment of Serious Adverse Events and other safety related events

Not applicable.

10.2.2 Reporting of (Serious) Adverse Events and other safety related events

Not applicable.

10.2.3 Follow up of (Serious) Adverse Events

Not applicable.

10.3 Medical Device Category A studies

10.3.1 Definition and Assessment of safety related events

Not applicable

10.3.2 Reporting of Safety related events

Not applicable.

11. STATISTICAL METHODS

11.1 Hypothesis

Specific immune responses against porins are generated after the administration of Vivotif® to healthy volunteers.

H_1: The median difference of specific immune responses after and before immunization is not zero.

H_0: The median difference of specific immune responses after and before immunization is zero.

11.2 Determination of Sample Size

This study is a pilot study; therefore, no sample size calculation was done. Fifteen participants in the Vivotif® group were considered sufficient to evaluate the methods and obtain meaningful results with respect to the induction of specific immune responses. Five untreated participants will serve as control.

11.3 Statistical criteria of termination of trial

The investigator may withdraw a participant from the trial for the following reasons:

- violation of the criteria for inclusion and/or
- Non-compliance of the subject (cf. 8.4).

11.4 Planned Analyses

11.4.1 Datasets to be analysed, analysis populations

In the experimental group, all subjects who received all three doses of vaccine will be included in the study analysis.

All subjects randomized to the control group will be included in the analysis.

11.4.2 Primary Analysis

The primary analysis of this study will focus on the determination of the antibody levels of IgM and IgG specific against porins in serum and stool, the number of porins-specific T cells from blood and the amount of porins-specific B cells in blood. Descriptive statistics will be shown separately for both treatment groups and will include the mean, sd, median, first and third quartile, minimum, and maximum for all time points. Differences to baseline will be investigated separately for both groups with a Wilcoxon signed rank test. If the distribution of the paired differences is not symmetric, the data may need to be transformed to produce symmetry.

11.4.3 Secondary Analyses

In the secondary analyses, the proportion of mutants from the original inoculum of live attenuated bacteria (Vivotif® vaccine) will be evaluated. The mean, sd, median, first and third quartile, minimum and maximum will be shown for all time points.

A Mann-Whitney *U* tests will be used to compare treated and untreated participants with respect to immune responses at baseline. Moreover, the mean immune response for visits 5-7 will be computed for each participant and compared between treated and untreated participants with a Mann-Whitney *U* test.

11.4.4 Interim analyses

The risk category of the present study is A. Safety and efficacy of Vivotif® live attenuated vaccine has been demonstrated in several clinical studies (please refer to section 3.4). Therefore, no interim

analysis will be performed in this study.

11.4.5 Safety analysis

Not applicable.

11.4.6 Deviation(s) from the original statistical plan

Deviations from the original statistical plan will be reported as non-substantial amendments.

11.5 Handling of missing data and drop-outs

An available data analysis will be performed, thus, missing data will not be imputed. A common problem in clinical trials is the missing data that occurs when patients do not complete the study and drop out without further measurements. Missing data cause the usual statistical analysis of complete or all available data to be subject to bias. There are no universally applicable methods for handling missing data. In this study the following measures will be taken:

(1) Reasons for dropouts and proportions for each treatment group will be reported. Characteristics for participants who did or did not drop-out of the study will be compared to determine if there are any clinically relevant differences between the two groups.

(2) Careful conduction of the study will be made to minimize the chance of dropouts.

(3) Post-dropout data will be collected on the primary endpoints, if at all possible.

(4) Depending on the time frame of the study drop-outs could be replaced, with previous authorization from the investigator and the sponsor.

12. QUALITY ASSURANCE AND CONTROL

The Sponsor is responsible for implementing and maintaining quality assurance and quality control systems with written Standard Operating Procedures (SOPs). The PI is responsible for proper training of all involved study personnel.

12.1 Data handling and record keeping / archiving

The sponsor-investigator must maintain adequate and accurate records to enable the conduct of the study to be fully documented, and the study data to be subsequently verified. These documents encompass the patient specific clinical source documents and the documents to be maintained in the investigator site file (ISF). These documents (except for the source documents) are to be maintained in the Trial Master File (TMF).

The investigator site file will contain the study protocol and any amendments, safety data of the study drugs, financial agreement, eCRFs together with all data changes made, EC and if applicable Health Authority approval with correspondence, all approved patient informed consent forms and signed patient informed consents, screening- and enrolment logs, patient identification list, drug records, safety reports, staff curricula vitae and authorization forms, and other appropriate documents/correspondence in accordance with ICH GCP and local regulations. All trial-related pertinent data must be maintained and filed by the investigator or his/her delegate in the ISF and kept at the study site at a secure place.

The investigator must ensure that patient anonymity is maintained, this means that patient identity should be protected, nevertheless re-identification only by the study site is possible, i.e. patient data is pseudonymized. On any document submitted to the sponsor, patients must be identified only by number, and never by name. The investigator must keep a patient identification code list showing the unique patient number (UPN), the patient's name, date of birth and address or any other locally accepted identifiers. Documents identifying the patients (e.g., signed informed consent forms) should not be sent to the sponsor, and must be kept in strict confidence by the investigator.

12.1.1 Case Report Forms

Case Report Form data will be captured via electronic data capture using the SecuTrial® system, a web based tool provided by Active Systems GmbH and supported by the data management of the monitoring institution. For each enrolled study participant a CRF will be maintained. CRFs will be kept current to reflect subject status at each phase during the course of study. Participants will not be identified in the CRF by name or initials and birth date. An appropriate coded identification (unique study identification number or UPN) will be used. On all study documents, other than the signed consent, and on all biological samples, the participant will be referred to only by the unique study identification number. Only study personnel are authorized to perform and access CRF entries. Any authorised person is electronically identified.

The investigator and site staff will enter and edit the data via a secure network, with secure access features (username, password and secure identification – an electronic password system). The investigator will have access to the data throughout the trial life cycle. The electronic Case Report Forms (eCRF) must be kept current to reflect patient status at any time point during the course of the trial. The investigator will approve the data using an electronic signature, and this approval is used to confirm accuracy of the data recorded.

12.1.2 Specification of source documents

Source data will be available at the site to document the existence of the study participants. Source data include the original documents relating to the study, as well as the medical treatment and medical

history of the participant. In the present study, source documents include, but are not limited to: visit dates, participation in study, Informed Consent Forms, SAEs, concomitant medication and results of relevant examinations. At the investigation site, source data will be stored in a secure location separate from the study participant identification information.

12.1.3 Record keeping / archiving

Copies of the electronic CRFs together with all data changes made will be supplied to the investigator at the end of the trial. The investigator will be responsible for retaining all records pertaining to the trial as specified in the appropriate contract, but must at least archive the records for a minimum of 10 years after study termination or premature termination of the clinical trial in a secure, lockable place.

Should the investigator wish to assign the study records to another party, or move them to another location, then the sponsor-investigator must be notified in advance. When the source documents are required for the continued care of the patient, appropriate copies should be made for storing off site.

12.2 Data management

12.2.1 Data Management System

The validated electronic data management system (SecuTrial®) will be used for study data recording. SecuTrial® is fully compliant with the regulatory requirements of ICH-GCP and FDA 21 CRF Part 11.

The electronic database will be provided and maintained by the data management of the CTU Kantonsspital St. Gallen. Prior to trial start and prior to any update release, the eCRF database is tested and validated by the data manager, the sponsor-investigator and/or his delegates.

12.2.2 Data security, access and back-up

The investigator and site staff will enter and edit the data via a secure network, with secure access features (username, password and secure identification – an electronic password system). 128 bit Secure Sockets Layer (SSL) data encryption is applied for data transfer via internet to ensure data security from outside. A complete electronic audit trail will be maintained.

12.2.3 Analysis and archiving

After the database has been declared complete and accurate it will be locked. Any changes to the database after that time may only be made by joint written agreement between the sponsor-investigator and the trial statistician.

After database closure, the data will be extracted and exported for analysis using appropriate software by the trial statistician. The final analysis of the data will only be performed after all queries have been resolved. Archiving is described in section 12.1.3.

12.2.4 Electronic and central data validation

While entering the data, the investigator will be prompted by logical checks (error messages) built into the web-based data entry screens performed on the data. Additional data review will be processed in parallel by the sponsor team, to look for unexpected patterns in data. If problematic data is detected, a query specifying problem and requesting clarification will be issued, and will be visible to the investigator via the eCRF. The investigators will then respond and clarify directly in the eCRF. This process will continue until database closure.

12.3 Monitoring

A quality visit will be performed during the course of the study. On request, access must be permitted to all source documents needed to verify the entries on the eCRF and other protocol-related documents, provided that patient confidentiality is maintained in accordance with local regulations.

12.4 Audits and Inspections

Authorised representatives of the Sponsor-Investigator may conduct audits of clinical research activities in accordance with internal standard operating procedures (SOPs) to evaluate compliance with the principles of GCP- and ICH-related guidelines.

Ethics committees may also wish to conduct an inspection (during the study or after its completion). Should an inspection be requested by an Ethics Committee, the investigator must inform the sponsor-investigator immediately that such a request has been made.

The investigator must permit such audits or inspections, and must facilitate them by providing access to the study related data and documents.

12.5 Confidentiality, Data Protection

Access to source documents will be permitted for purposes of audits and inspections and to the monitor as well during the quality visit (12.4) (ICHE6, 6.10). Personnel involved in the clinical trial (please refer to section 1) will have access to the protocol, dataset, statistical code during and after the study (publication, dissemination).

12.6 Storage of biological material and related health data

Biological samples will be stored for one year after termination of the study only with the participants consent independent from the study. Blood samples will be stored at -80°C in the facilities of the Institute of Immunobiology, Kantonsspital St Gallen. Stool samples will be stored at -80°C in the facilities of the Institute of Microbiology, ETH Zurich.

13. PUBLICATION AND DISSEMINATION POLICY

Data obtained from this study will be published in international scientific journals and will be presented at scientific meetings. The chairman and the sponsor-investigator will have ultimate authority over any of the activities related to the publication of the study results; study data may not be published or presented by third parties without their prior approval. Participants will not be identified in any publication by name, name initials or birth date, meaning that complete anonymity of participants will be ensured.

14. FUNDING AND SUPPORT

14.1 Funding

Financial support for this clinical trial is provided by the following foundations:

- Gottfried und Julia Bangerter-Rhyner
- Kantonsspital St. Gallen

14.2 Other Support

Not applicable

15. INSURANCE

For clinical trials of risk category A, an appropriate insurance for study participants is provided automatically by the Kantonsspital St. Gallen.

16. REFERENCES

1. Chen, H. M., Y. Wang, L. H. Su, and C. H. Chiu. 2013. Nontyphoid salmonella infection: microbiology, clinical features, and antimicrobial therapy. *Pediatr. Neonatol.* 54: 147-152.

2. Majowicz, S. E., J. Musto, E. Scallan, F. J. Angulo, M. Kirk, S. J. O'Brien, T. F. Jones, A. Fazil, and R. M. Hoekstra. 2010. The global burden of nontyphoidal Salmonella gastroenteritis. *Clin. Infect. Dis.* 50: 882-889.

3. Ochiai, R. L., C. J. Acosta, M. C. Danovaro-Holliday, D. Baiqing, S. K. Bhattacharya, M. D. Agtini, Z. A. Bhutta, d. G. Canh, M. Ali, S. Shin, J. Wain, A. L. Page, M. J. Albert, J. Farrar, R. Abu-Elyazeed, T. Pang, C. M. Galindo, S. L. von, and J. D. Clemens. 2008. A study of typhoid fever in five Asian countries: disease burden and implications for controls. *Bull. World Health Organ* 86: 260-268.

4. World Health Organisation. 2008. Typhoid vaccine: WHO position paper. *Weekly Epidemiological Record* .

5. Guzman, C. A., S. Borsutzky, M. Griot-Wenk, I. C. Metcalfe, J. Pearman, A. Collioud, D. Favre, and G. Dietrich. 2006. Vaccines against typhoid fever. *Vaccine* 24: 3804-3811.

6. Hessel, L., H. Debois, M. Fletcher, and R. Dumas. 1999. Experience with Salmonella typhi Vi capsular polysaccharide vaccine. *Eur. J. Clin. Microbiol. Infect. Dis.* 18: 609-620.

7. MacLennan, C. A., E. N. Gondwe, C. L. Msefula, R. A. Kingsley, N. R. Thomson, S. A. White, M. Goodall, D. J. Pickard, S. M. Graham, G. Dougan, C. A. Hart, M. E. Molyneux, and M. T. Drayson. 2008. The neglected role of antibody in protection against bacteremia caused by nontyphoidal strains of Salmonella in African children. *J. Clin. Invest* 118: 1553-1562.

8. Ortiz, V., A. Isibasi, E. Garcia-Ortigoza, and J. Kumate. 1989. Immunoblot detection of class-specific humoral immune response to outer membrane proteins isolated from Salmonella typhi in humans with typhoid fever. *J. Clin. Microbiol.* 27: 1640-1645.

9. Salazar-Gonzalez, R. M., C. Maldonado-Bernal, N. E. Ramirez-Cruz, N. Rios-Sarabia, J. Beltran-Nava, J. Castanon-Gonzalez, N. Castillo-Torres, J. A. Palma-Aguirre, M. Carrera-Camargo, C. Lopez-Macias, and A. Isibasi. 2004. Induction of cellular immune response and anti-Salmonella enterica serovar typhi bactericidal antibodies in healthy volunteers by immunization with a vaccine candidate against typhoid fever. *Immunol. Lett.* 93: 115-122.

10. MacLennan, C. A., J. J. Gilchrist, M. A. Gordon, A. F. Cunningham, M. Cobbold, M. Goodall, R. A. Kingsley, J. J. van Oosterhout, C. L. Msefula, W. L. Mandala, D. L. Leyton, J. L. Marshall, E. N. Gondwe, S. Bobat, C. Lopez-Macias, R. Doffinger, I. R. Henderson, E. E. Zijlstra, G. Dougan, M. T. Drayson, I. C. MacLennan, and M. E. Molyneux. 2010. Dysregulated humoral immunity to nontyphoidal Salmonella in HIV-infected African adults. *Science* 328: 508-512.

11. Secundino, I., C. Lopez-Macias, L. Cervantes-Barragan, C. Gil-Cruz, N. Rios-Sarabia, R. Pastelin-Palacios, M. A. Villasis-Keever, I. Becker, J. L. Puente, E. Calva, and A. Isibasi. 2006. Salmonella porins induce a sustained, lifelong specific bactericidal antibody memory response. *Immunology* 117: 59-70.

12. Cervantes-Barragan, L., C. Gil-Cruz, R. Pastelin-Palacios, K. S. Lang, A. Isibasi, B. Ludewig, and C. Lopez-Macias. 2009. TLR2 and TLR4 signaling shapes specific antibody responses to Salmonella typhi antigens. *Eur. J. Immunol.* 39: 126-135.

13. Germanier, R., and E. Fuer. 1975. Isolation and characterization of Gal E mutant Ty 21a of Salmonella typhi: a candidate strain for a live, oral typhoid vaccine. *J. Infect. Dis.* 131: 553-558.

14. Germanier, R., and E. Furer. 1983. Characteristics of the attenuated oral vaccine strain "S. typhi" Ty 21a. *Dev. Biol. Stand.* 53: 3-7.

15. Miller S.I., E. L. H. D. A. P. 1995. Salmonella. In *Principles and practice of infectious diseases*, 4th ed. J. E. B. R. D. G.L.Mandell, ed. Churchill Livingstone Inc. 2013-2033.

16. 1994. Recommendations of the Advisory Committee on Immunization Practices (ACIP): Typhoid Immunization. MMWR.

17. Ames, W. R., and M. Robins. 1943. Age and Sex as Factors in the Development of the Typhoid Carrier State, and a Method for Estimating Carrier Prevalence. *Am. J. Public Health Nations. Health* 33: 221-230.

18. Wahdan, M. H., C. Serie, Y. Cerisier, S. Sallam, and R. Germanier. 1982. A controlled field trial of live Salmonella typhi strain Ty 21a oral vaccine against typhoid: three-year results. *J. Infect. Dis.* 145: 292-295.

19. Black, R. E., M. M. Levine, C. Ferreccio, M. L. Clements, C. Lanata, J. Rooney, and R. Germanier. 1990. Efficacy of one or two doses of Ty21a Salmonella typhi vaccine in enteric-coated capsules in a controlled field trial. Chilean Typhoid Committee. *Vaccine* 8: 81-84.

20. Levine, M. M., C. Ferreccio, R. E. Black, and R. Germanier. 1987. Large-scale field trial of Ty21a live oral typhoid vaccine in enteric-coated capsule formulation. *Lancet* 1: 1049-1052.

21. Levine, M. M., C. Ferreccio, R. E. Black, C. O. Tacket, and R. Germanier. 1989. Progress in vaccines against typhoid fever. *Rev. Infect. Dis.* 11 Suppl 3: S552-S567.

22. Ferreccio, C., M. M. Levine, H. Rodriguez, and R. Contreras. 1989. Comparative efficacy of two, three, or four doses of TY21a live oral typhoid vaccine in enteric-coated capsules: a field trial in an endemic area. *J. Infect. Dis.* 159: 766-769.

23. Simanjuntak, C. H., F. P. Paleologo, N. H. Punjabi, R. Darmowigoto, Soeprawoto, H. Totosudirjo, P. Haryanto, E. Suprijanto, N. D. Witham, and S. L. Hoffman. 1991. Oral immunisation against typhoid fever in Indonesia with Ty21a vaccine. *Lancet* 338: 1055-1059.

24. Gilman, R. H., R. B. Hornick, W. E. Woodard, H. L. DuPont, M. J. Snyder, M. M. Levine, and J. P. Libonati. 1977. Evaluation of a UDP-glucose-4-epimeraseless mutant of Salmonella typhi as a liver oral vaccine. *J. Infect. Dis.* 136: 717-723.

25. Data on file. 2014. Swiss Serum and Vaccine Institute Berne. Switzerland.
